# Net greenhouse gas balance of fibre wood plantation on peat in Indonesia

**DOI:** 10.1038/s41586-023-05860-9

**Published:** 2023-04-05

**Authors:** Chandra S. Deshmukh, Ari P. Susanto, Nardi Nardi, Nurholis Nurholis, Sofyan Kurnianto, Yogi Suardiwerianto, M. Hendrizal, Ade Rhinaldy, Reyzaldi E. Mahfiz, Ankur R. Desai, Susan E. Page, Alexander R. Cobb, Takashi Hirano, Frédéric Guérin, Dominique Serça, Yves T. Prairie, Fahmuddin Agus, Dwi Astiani, Supiandi Sabiham, Chris D. Evans

**Affiliations:** 1 Asia Pacific Resources International Ltd., Pelalawan Regency, Indonesia; 2 Department of Atmospheric and Oceanic Sciences, University of Wisconsin-Madison, Madison, WI USA; 3 School of Geography, Geology and the Environment, University of Leicester, Leicester, UK; 4 Singapore-MIT Alliance for Research and Technology, Singapore, Singapore; 5 Research Faculty of Agriculture, Hokkaido University, Sapporo, Japan; 6 Géosciences Environnement Toulouse, CNRS, IRD, Université Paul-Sabatier, Toulouse, France; 7 LAERO, Université de Toulouse, CNRS, IRD, UT3, Toulouse, France; 8 UNESCO Chair in Global Environmental Change, Université du Québec à Montréal, Montréal, Québec Canada; 9 National Research and Innovation Agency (BRIN), Cibinong, Indonesia; 10 Faculty of Forestry, Tanjungpura University, Pontianak, Indonesia; 11 Department of Soil Science and Land Resources, IPB University, Bogor, Indonesia; 12 UK Centre for Ecology & Hydrology, Bangor, UK

**Keywords:** Carbon cycle, Climate-change mitigation, Governance

## Abstract

Tropical peatlands cycle and store large amounts of carbon in their soil and biomass^1–5^. Climate and land-use change alters greenhouse gas (GHG) fluxes of tropical peatlands, but the magnitude of these changes remains highly uncertain^6–19^. Here we measure net ecosystem exchanges of carbon dioxide, methane and soil nitrous oxide fluxes between October 2016 and May 2022 from *Acacia crassicarpa* plantation, degraded forest and intact forest within the same peat landscape, representing land-cover-change trajectories in Sumatra, Indonesia. This allows us to present a full plantation rotation GHG flux balance in a fibre wood plantation on peatland. We find that the *Acacia* plantation has lower GHG emissions than the degraded site with a similar average groundwater level (GWL), despite more intensive land use. The GHG emissions from the *Acacia* plantation over a full plantation rotation (35.2 ± 4.7 tCO_2_-eq ha^−1^ year^−1^, average ± standard deviation) were around two times higher than those from the intact forest (20.3 ± 3.7 tCO_2_-eq ha^−1^ year^−1^), but only half of the current Intergovernmental Panel on Climate Change (IPCC) Tier 1 emission factor (EF)^20^ for this land use. Our results can help to reduce the uncertainty in GHG emissions estimates, provide an estimate of the impact of land-use change on tropical peat and develop science-based peatland management practices as nature-based climate solutions.

## Abstract (web summary)

Measurements of the net ecosystem exchanges of CO_2_, CH_4_ and soil N_2_O from *Acacia* plantation, degraded forest and intact forest enable presentation of the peatland wood plantation rotation greenhouse gas flux balance.

## Main

Over the Holocene, tropical peatlands have accumulated at least 75 Gt of carbon (C) in partially decomposed debris (wood, roots, litter, leaves) under waterlogged anoxic environments^1–5^. A fine balance between hydrology, ecology and landscape morphology has resulted in this long-term C store^6–8^. Climate and other environmental changes are, however, affecting this C store as a result of warming, drying conditions and change in disturbance rates^9–17^. Particularly, decreased rainfall, increased seasonality and frequent days without rainfall are resulting in GWL drawdowns^7,12,13^, which cause C loss^14–17^.

Tropical peatlands are among the world’s most threatened ecosystems owing to land demand driven by population growth and economic development^21^. In Southeast Asia, which hosts at least one-third of the total tropical peatlands^3,4^, most peatland conversion has occurred since the late 1990s^21^. A total peatland area of 7.8 million hectares is managed for agriculture and silviculture, of which more than one million hectares are under fibre wood (mostly *A. crassicarpa*) plantations^21^. Artificial GWL drawdown in agriculture and plantations on peatland exposes previously accumulated peat organic material to oxygen and promotes aerobic decomposition of organic C, resulting in carbon dioxide (CO_2_) emissions^22,23^ and associated land subsidence^24–26^. At present, the IPCC Tier 1 CO_2_ EF^20^ for short-rotation tree plantations on tropical peat is entirely based on the use of short-term measurements from the 3–8 years after drainage using subsidence^22^ and soil-chamber^23^ techniques. Furthermore, tropical peatlands emit methane (CH_4_)^27,28^ and nitrous oxide (N_2_O)^17,29^, potent GHGs, yet assessments of the contributions made by these gases to the full peatland GHG balance are scarce^20^. Existing estimates of GHG emissions from tropical peatlands continue to be debated^20,30^ with large observed variability (0.04–2.79 GtCO_2_-eq year^−1^)^18^ and resulting uncertainty^19^.

From a climate-forcing perspective, the effects of a land-use change on the atmospheric GHG concentrations (that is, the extra GHG fluxes that the atmosphere will see because of current land use) will be determined by the change in emissions relative to those occurring before the land-use change^17,31^. Despite the increasing awareness of the significance of GHG fluxes from managed peatlands, there have been few experimental studies evaluating the GHG balance before and after a land-use change has occurred. Thus, a better quantitative and process-based knowledge of how the tropical peat C store responds to land-use change under current climate conditions is an urgent area of enquiry that can inform strategies for responsible peatland management^32^ under national and global frameworks of climate change.

**Fig. 1:**
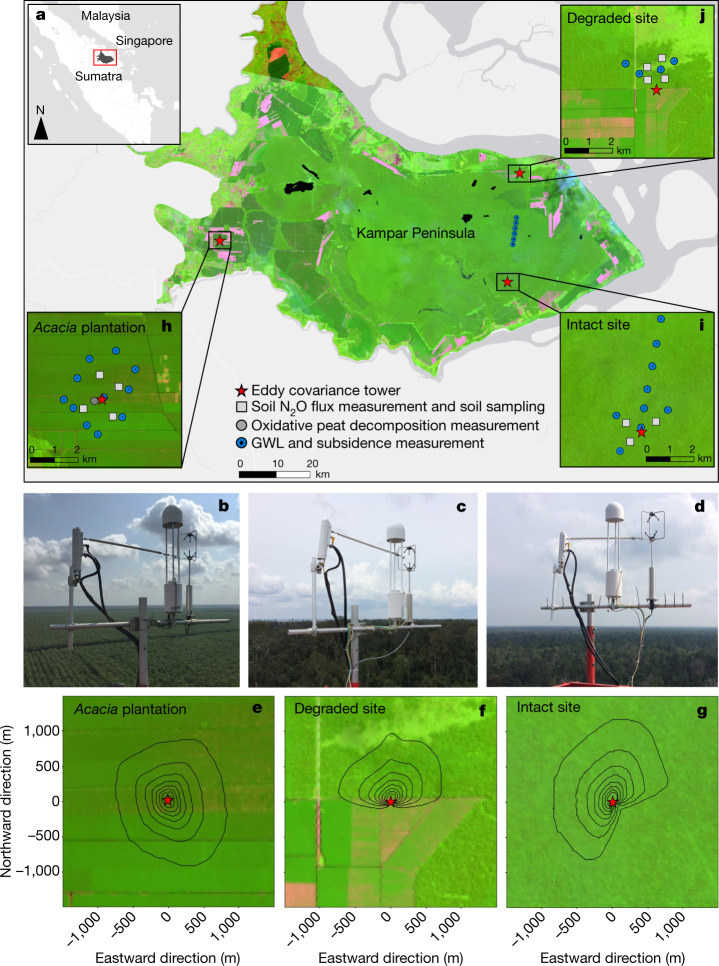
Location of the study area, Kampar Peninsula in Sumatra, Indonesia. **a**, Location of research sites with satellite images from Landsat 8 (source: https://earthexplorer.usgs.gov/). Photographs of the eddy covariance instruments installed at the top of the tower at *Acacia* plantation (**b**), degraded site (**c**) and intact site (**d**). For detailed site information, see Methods. Integrated eddy covariance footprint contour lines from 10% to 80% in 10% intervals over *Acacia* plantation for October 2016–May 2021 (**e**), degraded site for October 2016–May 2022 (**f**) and intact site for June 2017–May 2022 (**g**). GWL, peat subsidence, oxidative peat decomposition, soil N_2_O flux and soil-sampling locations at *Acacia* plantation (**h**), intact site (**i**) and degraded site (**j**). An integrated climatologic footprint analysis indicated that approximately 80% of fluxes originated within 1,000 m in the upwind direction of each tower. Esri, HERE, Garmin, (c) OpenStreetMap contributors and the GIS user community.

**Fig. 2:**
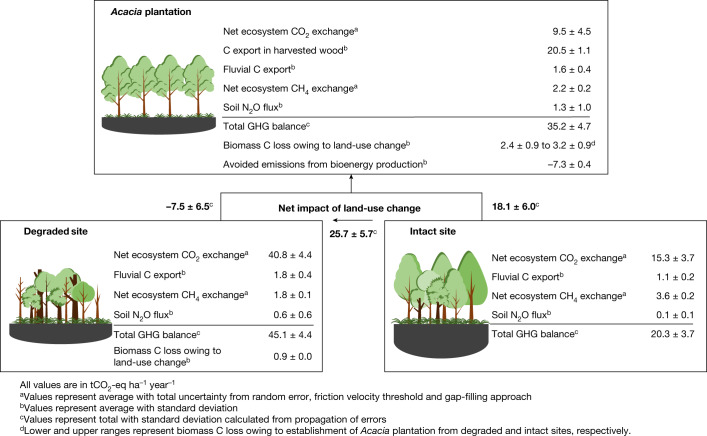
GHG balance of *Acacia* plantation, degraded and intact peat swamp forest in Sumatra, Indonesia. To quantify total GHG balance in carbon dioxide equivalent (CO_2_-eq), we used a sustained-flux global-warming potential (SGWP) of 1, 45 and 270 for CO_2_, CH_4_ and N_2_O, respectively, over a 100-year time period^39^. Total GHG balance = (net ecosystem CO_2_ exchange + net ecosystem CH_4_-C exchange + fluvial C export + C export in harvested wood, where applicable) + (net ecosystem CH_4_ exchange × SGWP) + (soil N_2_O flux × SGWP). We assumed that all fluvial C export is ultimately converted to CO_2_ (ref. ^38^). Avoided emissions from bioenergy production are calculated by assuming that 54% of harvested wood is used for bioenergy production (details in Supplementary Methods). The bold numbers indicate net impact of land-use change. Positive values indicate emission to the atmosphere and negative values indicate avoided emission.

This study represents, to the best of our knowledge, the first GHG balance investigation undertaken in any fibre wood plantation on peatland (and indeed any soil type) globally to cover a full plantation rotation and all major GHG flux terms, including biomass C loss owing to plantation establishment, C export in harvested wood and fluvial C exports. We compare the GHG balance at the *Acacia* plantation with more than 5 years of measurements at the degraded site and 5 years of measurements at the intact site (Figs. 1 and 2 and Extended Data Table 1).

## CO_2_ flux

**Extended Data Fig. 1:**
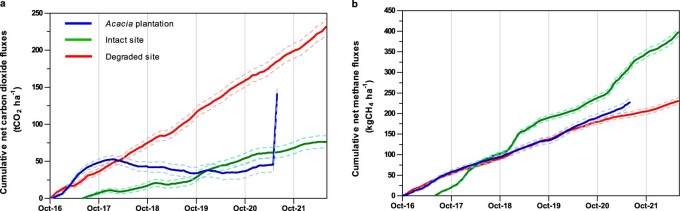
*Acacia* plantation, degraded and intact peat swamp forest in Sumatra, Indonesia, are emitting CO_2_ and CH_4_ to the atmosphere. Cumulative measured net CO_2_ (**a**) and net CH_4_ (**b**) fluxes with cumulative flux uncertainty (random error, friction velocity threshold and gap-filling approach) at the *Acacia* plantation (blue), degraded site (red) and intact site (green). Carbon export in harvested wood at the *Acacia* plantation is added in the end of plantation rotation, conservatively assuming that all harvested C would be returned to the atmosphere as CO_2_. Intact and degraded sites were considered to have had no biomass C export during the study period. Positive values indicate emission to the atmosphere.

Over a 4.7-year period encompassing one full *Acacia* plantation rotation (the fourth rotation during 17–22 years after drainage), the average GWL was −0.65 ± 0.17 m, for which a negative GWL indicates that the water level was below the peat surface (Extended Data Table 2). Net ecosystem exchanges of CO_2_ (NEE-CO_2_; net gaseous CO_2_ exchange between ecosystem and atmosphere) varied with plantation age; it was highest (48.4 ± 4.7 tCO_2_ ha^−1^ year^−1^) in the first year after planting, lowest (−8.8 ± 4.5 tCO_2_ ha^−1^ year^−1^) in the third year with highest tree growth and then rose again to 11.7 ± 6.0 tCO_2_ ha^−1^ year^−1^ before harvesting (Extended Data Fig. 1 and Extended Data Table 3) (for which positive NEE-CO_2_ values indicate net CO_2_ emissions and negative values indicate net CO_2_ uptake). The substantial net CO_2_ emissions during the early stage of the plantation were mainly because of the low photosynthetic rates of the young trees and also potentially driven by the decomposition of organic matter from harvested residues (that is, leaves, branches, bark, roots and stumps) from the previous plantation rotation. After canopy closure, the emissions from oxidative peat decomposition (Extended Data Table 3) were largely outbalanced by high rates of photosynthesis and C fixation (Extended Data Fig. 1). Over the plantation rotation, the average NEE-CO_2_ was 9.5 ± 4.5 tCO_2_ ha^−1^ year^−1^ (Fig. 2 and Extended Data Table 4). The average peat subsidence rate was −3.0 ± 0.9 cm year^−1^ (Extended Data Table 5) (negative peat subsidence indicates that the ground surface elevation was falling). The C export in harvested wood was 26.3 ± 1.4 tC ha^−1^, corresponding to 96.5 ± 5.2 tCO_2_ ha^−1^ (20.5 ± 1.1 tCO_2_ ha^−1^ year^−1^ when annualized over the plantation rotation; Fig. 2 and Extended Data Table 4). Thus, the sum of NEE-CO_2_ and C export in harvested wood indicates that the *Acacia* plantation functioned as a CO_2_ source of 30.0 ± 4.6 tCO_2_ ha^−1^ year^−1^ over the plantation rotation (Fig. 2 and Extended Data Table 4). The observed net CO_2_ emissions can be attributed to peat aeration owing to a consistently deep GWL, which enhances heterotrophic respiration rates, combined with a higher soil temperature (intact site = 27.5 ± 0.5 °C versus *Acacia* site = 29.3 ± 1.0 °C; Extended Data Table 2) owing to both canopy-cover loss and GWL drawdown, which further boosts microbial activities and heterotrophic respiration. Note that this calculation conservatively assumed that all harvested C would be returned to the atmosphere as CO_2_, as harvested wood was used to produce bioenergy and pulp products, which is common practice for these types of forest plantation.

We also calculated avoided emissions of 7.3 ± 0.4 tCO_2_-eq ha^−1^ year^−1^, resulting from the use of tree biomass for bioenergy (see Supplementary Methods), in place of coal burning that would otherwise have been used to support pulp mill operations (Fig. 2 and Extended Data Table 4). This avoided emission through bioenergy production as a by-product of the pulp manufacturing process could be considered to partly offset emissions from the plantation itself, although it clearly does not negate the peat CO_2_ emission.

In degraded peat swamp forest, the GWL was consistently low throughout the study period at the degraded site, with an average of −0.69 ± 0.18 m (Extended Data Table 2). NEE-CO_2_ did not show clear seasonal and interannual variability. The degraded site emitted 40.8 ± 4.4 tCO_2_ ha^−1^ year^−1^ and subsided −3.6 ± 1.2 cm year^−1^ (Fig. 2 and Extended Data Table 3), consistent with previous observations in ref. ^17^. The observed large CO_2_ emissions can be attributed to peat aeration owing to a consistently deep GWL as described for the *Acacia* site. Coarse woody debris from fallen dead trees may also have contributed to the CO_2_ emissions, as fallen trees do not decompose instantaneously, providing a lagged but sustained contribution to CO_2_ emissions.

In intact peat swamp forest, the GWL followed the seasonal and interannual variability in rainfall (Extended Data Table 2), in line with the initial measurements in ref. ^17^. The GWL remained below the peat surface for >80% of the study period, indicating that a substantial part of the upper peat profile was aerated. NEE-CO_2_ showed strong seasonal and interannual patterns corresponding to the GWL fluctuation (Extended Data Fig. 1). The results indicate that large net CO_2_ emissions during dry seasons were not entirely balanced by relatively small net CO_2_ uptake during the wet seasons (Extended Data Fig. 1). Over a 5-year measurement period, the annual NEE-CO_2_ ranged from 9.1 ± 3.7 to 25.6 ± 4.1 tCO_2_ ha^−1^ year^−1^, with an average value of 15.3 ± 3.7 tCO_2_ ha^−1^ year^−1^ (Extended Data Table 3). The CO_2_ emissions owing to GWL drawdown are consistent with previous studies in tropical peatlands in which reduced peat accumulation rates^8,15^, a hiatus in peat genesis^33^ or even C loss^14–17^ have been reported in response to droughts driven by intense and frequent El Niño–Southern Oscillation activity.

**Extended Data Fig. 2:**
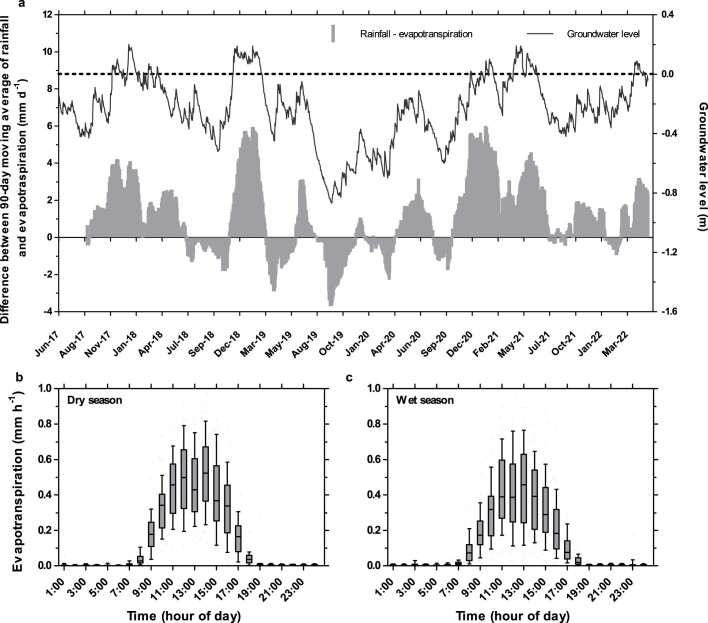
GWL is controlled by the balance between rainfall and evapotranspiration at intact peat swamp forest in Sumatra, Indonesia. **a**, Time series of average GWL from three piezometers spanning 12 km with difference between 90 days moving average of rainfall and evapotranspiration. Negative difference indicates rainfall deficit. Positive and negative GWL values indicate the water level above and below the peat hollow surface, respectively. Diel pattern of evapotranspiration during dry season (February and July) (**b**) and wet season (April and November) (**c**) over the measurement periods show negligible evapotranspiration in the nighttime. The boxes show the median value and the interquartile range and whiskers denote the full range.

The evapotranspiration measurements clearly indicate that actual daily evapotranspiration (4.2 mm day^−1^) exceeded daily rainfall for around 76% of the study period (Extended Data Table 2). Notably, we observed more than 220 days without rainfall every year (Extended Data Table 2). During days without rainfall, the GWL would recede at an average rate of 10.3 mm day^−1^, resulting from the seepage and evapotranspiration in this ombrotrophic environment. The seepage rates owing to groundwater and subsurface flows, as calculated from GWL drawdown between midnight and 06:00 local time (when evapotranspiration is negligible; Extended Data Fig. 2), was 1.4 mm during a single 12-h night (that is, 2.8 mm day^−1^), which is similar to a pristine tropical peatland in Brunei^7^. The evapotranspiration resulted in GWL drawdown of 7.5 mm day^−1^ during days without rainfall at our study site. During prolonged drought periods induced by climate extremes in 2019, when we observed only 45 mm rainfall during a consecutive 90-day period, the GWL fell to below −0.70 m, resulting in a large peat surface drop of −7.0 ± 1.3 cm in the intact sites (Extended Data Table 5). The peat surface had not rebounded from the 2019 perturbation by the end of the record, resulting in a total subsidence of −7.1 ± 2.4 cm during the period December 2017–May 2022 (Extended Data Table 5).

The close link between net rainfall (total rainfall minus evapotranspiration) and GWL (Extended Data Fig. 2) confirms that observed relatively low rainfall combined with increased seasonality and days without rainfall play a central role in shaping the seasonal and interannual variability of intact tropical peatland hydrology and therefore of CO_2_ fluxes^7^. The El Niño^34^ and positive Indian Ocean Dipole (IOD)^35^ observations indicate that the region has experienced several moderate to very strong drought events in the recent past, suggesting that tropical peatland ecosystems are exposing and responding to changes in rainfall regime, which may limit their role as a carbon sink.

Given that GWLs at our intact forest site were slightly lower (annual rainfall = 1,883 mm year^−1^; Extended Data Table 2) than those reported for a pristine peat swamp forest (annual rainfall = 2,880 mm year^−1^)^7^, we cannot entirely rule out some impact of surrounding land use on the hydrology and function of the peat dome as a whole. However, plantation water management is not believed to have affected forest hydrology at the flux tower footprint in the study area. Previous analysis suggested that such effects occurred within 300 m of the plantation boundary^25^, and recent multivariate analysis indicates that subsidence in the interior forest is independent of distance from plantation canals^26^. This is further indicated by subsidence at rates of −1.4 cm year^−1^ (Extended Data Table 5) observed at sampling locations between 7 to 10 km from the active plantation edge (Fig. 1). There was clearly a strong association during the study period between C loss, subsidence and droughts driven by regional climate extremes^26^. Our results indicate that even low-level or indirect human disturbance (for example, by means of climate change) can lead to C loss, highlighting the hydroclimatic vulnerability of C in forested tropical peatlands^13–16^.

## Other GHG fluxes and C loss

**Fig. 3:**
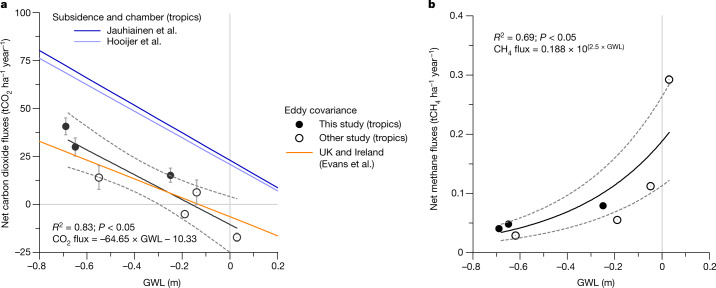
GWL controls carbon dioxide and methane fluxes in tropical peatlands. **a**, Carbon dioxide (CO_2_). **b**, Methane (CH_4_). Relationship between net CO_2_ and CH_4_ fluxes and GWL were derived from published eddy covariance flux studies in tropical peatlands. The solid lines show the best-fit models and the dashed lines show 95% confidence intervals. The statistical test used a significance level of 5%. Positive values indicate net emission to the atmosphere, negative values indicates net uptake by the ecosystem. CO_2_ results are compared with a previous relationship between CO_2_ fluxes and GWL derived from subsidence^22^ and soil flux chamber^23^ measurements and a relationship for peatlands in the United Kingdom and Ireland^45^ is based on eddy covariance measurements. Positive and negative GWL values indicate the water level above and below the peat hollow surface, respectively.

Net ecosystem exchanges of CH_4_ (NEE-CH_4_; net gaseous CH_4_ exchange between ecosystem and atmosphere) were positive at all sites, but lower in the *Acacia* plantation and degraded site than in the intact site (Fig. 2 and Extended Data Table 4), consistent with lower GWLs promoting methanotrophy in the aerobic zone^27^. GWL drawdown below the root zone will also limit plant-mediated transport of CH_4_ from the anaerobic zone to the atmosphere^27^. The finding that CH_4_ emissions remained positive despite low GWLs (Fig. 3) may be attributed to emissions from vegetation and water surfaces^27^.

**Extended Data Fig. 3:**
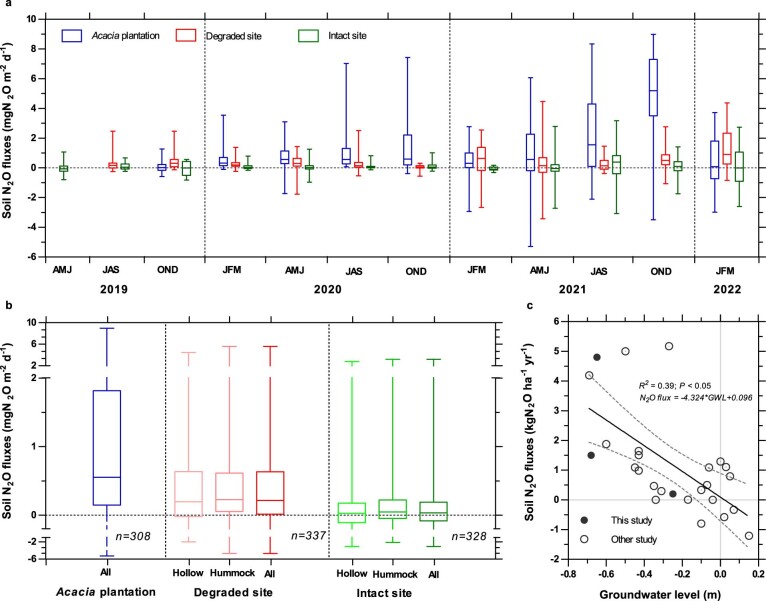
Soil N_2_O emissions increase as GWLs decrease in tropical peatlands. Temporal variation (**a**) and spatial variation (**b**) in soil N_2_O fluxes from *Acacia* plantation (blue), degraded site (red) and intact site (green). The boxes show the median value and the interquartile range and whiskers denote the full range of all chambers. The plus signs (+) in the boxes of panel **b** show the average values. The *n* values represent the total number of soil N_2_O flux measurements. **c**, Relationship between N_2_O fluxes and GWL were derived from soil flux chamber measurements on various land uses across tropical peatlands. Positive and negative GWL values indicate the water level above and below the peat surface, respectively. Positive flux value indicates emission to the atmosphere and negative value indicates uptake by the soil.

Soil N_2_O fluxes at the *Acacia* plantation were higher than at the degraded and intact sites (Fig. 2 and Extended Data Fig. 3), but were within the range of fluxes reported from oil palm plantation on peat^29,30^. Higher emissions from the plantation can be explained by a combination of leguminous *Acacia* trees that increase mineral nitrogen (N) availability through N fixation; accelerated mineralization of the peat under aerobic conditions (Extended Data Fig. 3) releasing mineral N as ammonium (Extended Data Table 1) and producing N_2_O and nitrate during the nitrification process; and high availability of labile C and nitrate from rapid fine-root turnover (Extended Data Table 1), providing a substrate for denitrifier heterotrophs.

A previous study^36^ within the same landscape reported fluvial C export of 0.3 ± 0.1 and 0.5 ± 0.1 tC ha^−1^ year^−1^ in the intact and degraded sites, respectively. Owing to lack of fluvial C-export measurements for the *Acacia* plantation, we used a value of 0.4 ± 0.1 tC ha^−1^ year^−1^ from a managed oil palm plantation in Southeast Asia^37^. Notably, fluvial C exports are fairly small compared with direct CO_2_ emissions. We conservatively assume that all fluvial C export is ultimately emitted as CO_2_ (ref. ^38^). The increased fluvial C export from the plantation and degraded forest may be attributed to enhanced mineralization with deeper GWL^37^.

Finally, the measured aboveground and belowground biomass C stock was highest in the intact forest (aboveground biomass = 105.6 ± 21.7 tC ha^−1^ and belowground biomass = 24.8 ± 5.1 tC ha^−1^) and decreased in the degraded forest (aboveground biomass = 88.7 ± 22.9 tC ha^−1^ and belowground biomass = 18.2 ± 4.7 tC ha^−1^) and the *Acacia* plantation (aboveground biomass = 35.2 ± 1.9 tC ha^−1^ and belowground biomass = 7.2 ± 0.4 tC ha^−1^, averaged over whole plantation rotation). Over a 100-year timescale (see Methods), biomass C losses owing to land-use change from intact forest were 0.9 ± 0.0 and 3.2 ± 0.9 tCO_2_ ha^−1^ year^−1^ in the degraded forest and *Acacia* plantation, respectively (Fig. 2 and Extended Data Table 4). Biomass C loss owing to plantation establishment on degraded forest was 2.4 ± 0.9 tCO_2_ ha^−1^ year^−1^ (Extended Data Table 4).

## Net GHG balance of *Acacia* plantation

Comparison of GHG fluxes at the *Acacia* plantation and degraded and intact sites in this tropical peat landscape indicates that conversion of intact forest to *Acacia* plantation or degraded forest results in a substantial increase in CO_2_ and N_2_O emissions and a decrease in CH_4_ emissions. Overall, the associated warming impact of higher CO_2_ and N_2_O emissions is larger than the accompanying cooling impact of lower CH_4_ emissions (Fig. 2). We calculated total GHG balances of all sites using a sustained-flux global-warming potential of 1, 45 and 270 for CO_2_, CH_4_ and N_2_O, respectively, over a 100-year time period^39^. The GHG balance and the subsidence rate in the *Acacia* plantation were around two times higher than those measured at the intact site (Fig. 2 and Extended Data Table 5). The measured CO_2_ emissions in this study indicate that the long-term rate of C accumulation of 2.8 tCO_2_ ha^−1^ year^−1^ in the Kampar Peninsula^8^ may no longer be occurring. If we take the measured GHG balance of the intact forest site as a reference, and treat our data from the fourth *Acacia* rotation as representative of longer-term conditions, then the conversion of intact site to *Acacia* plantation results in a long-term net increase in GHG emissions of 18.1 ± 6.0 tCO_2_-eq ha^−1^ year^−1^ (Fig. 2).

Our study is the first, to our knowledge, to provide an estimate of CO_2_ emissions from tropical *Acacia* plantation on peat based on the eddy covariance method, over a full plantation rotation. The CO_2_ EF is critical to GHG inventories in *Acacia* plantations, given that, in *Acacia* plantation, about 90% of peat on-site GHG emissions is released as CO_2_ (Fig. 2). We were able to reduce the uncertainties associated with variations in flux during the full plantation rotation and in biomass removal at the end of the rotation, which make estimating the average C balance of high-latitude peatlands with multidecadal rotations highly problematic. Our directly measured CO_2_ balance of the *Acacia* plantation is half of the IPCC Tier 1 EF value of 73 tCO_2_ ha^−1^ year^−1^. Two of the key studies^22,23^ used to derive the Tier 1 EF were carried out in the same plantation area in the initial 3–8 years after drainage, so interregional differences cannot explain the discrepancy. Subsidence rates^24,26,40^ and CO_2_ emissions^30,41,42^ in tropical peatlands are reported to decrease over time following drainage. An increase in bulk density (0.08 g cm^−3^ during 3–8 years after drainage in refs. ^22,23^ versus 0.20 g cm^−3^ during 17–22 years after drainage in this study; Extended Data Table 1), owing to peat compaction during land preparation, may result in lower peat oxidative decomposition because of the increase in soil water content and decrease in soil gas diffusivity^43^. Furthermore, a decline in soil organic matter quality^44^ and nutrient availability over time may leave behind a more stable peat matrix, resulting in a decrease in substrate-driven rates of CO_2_ production^41^ from peat decomposition. Finally, not all of the emissions in the initial years after plantation establishment^22,23^ would come directly from peat C decomposition, given that considerable forest biomass residues would also contribute to the initial CO_2_ loss, with most^42^ of the forest residues decomposing in the initial years after conversion.

Some other factors may have contributed to lower than expected long-term plantation emissions in our study. Improved water management practices, reflected in higher average GWL than reported in previous studies^22,23^, may have reduced oxidation rates to some extent. We also measured a C input to the peat of around 12 tCO_2_ ha^−1^ year^−1^ (calculated as the difference between the oxidative peat decomposition (41.7 tCO_2_ ha^−1^ year^−1^; Extended Data Table 3) and the sum of NEE-CO_2_ (9.5 tCO_2_ ha^−1^ year^−1^) and C export in harvested wood (20.5 tCO_2_ ha^−1^ year^−1^)) over the full plantation rotation. This C input, derived from litter, roots, stumps and bark residues, is not measured during chamber and subsidence studies, which may have led to overestimation of net CO_2_ emissions. Although our results confirm that fibre wood plantations are substantial net GHG sources, these results indicate that there may be opportunities to increase soil C input through better post-harvest residue management. Further research is needed to confirm the potential scale of increase in C input that could realistically be achieved.

The data from our three study sites, along with four other published eddy covariance studies from tropical peatlands (Extended Data Table 6), conform well to a linear relationship between CO_2_ flux and GWL (*R*^2^ = 0.83, *P* < 0.05; Fig. 3), suggesting that measured emissions are broadly consistent with those of other studies that applied a similarly rigorous whole-ecosystem eddy covariance measurement approach. As is evident from Fig. 3, net CO_2_ fluxes and their relationships with GWL derived from eddy covariance studies are substantially lower than those obtained from chamber and subsidence studies in the same ecosystems. Although the number of published eddy covariance studies from tropical peatlands remains insufficient to establish whether these differences are systematic, a similar offset is evident in CO_2_ flux versus GWL relationships derived from eddy covariance data^45^ and chamber data^46^ for high-latitude peatlands. Although further data are needed, we therefore tentatively conclude that emissions from *Acacia* plantation are substantially lower than the current IPCC Tier 1 EFs, as a result of methodological limitations to the data available at the time of publication of the IPCC Wetlands Supplement^20^ and changes in peat physicochemical properties with time since drainage.

Our results should not be extrapolated to other agriculture on peat in the region (for example, sago, oil palm, rubber plantations etc.) or to other tropical peatlands, such as those of the Amazon and Congo basins, because they have different rainfall regimes, vegetation and peat-formation histories. Nevertheless, the strong linear relationship between CO_2_ flux and GWL shown in Fig. 3 does suggest that, when the average annual GWL is known, the peatland CO_2_ balance can be predicted with some degree of confidence. This is in line with work^45,47^ on high-latitude peatlands suggesting that GWLs are more important than local climate or other management factors. Furthermore, although the relationship between CO_2_ flux and GWL is steeper for tropical peatlands compared with the full set of high-latitude flux tower data collated in ref. ^45^, we found less difference than expected between the tropical data and the data presented from the UK and Irish sites in the same study (Fig. 3). This finding is in marked contrast to a recently published synthesis study suggesting that tropical peatlands are inherently more sensitive to CO_2_ loss following GWL drawdown^48^. However, given that ref. ^48^ incorporated the same chamber studies used to derive the IPCC Tier 1 EF, we believe that it may have overestimated rates of CO_2_ loss from tropical peatlands for the same reasons noted above.

Using our EFs, net GHG emissions from *Acacia* plantations on peat in Indonesia are calculated to be 20 Mt CO_2_-eq year^−1^ (based on the area of *Acacia* plantation on peat in Indonesia, 1.12 × 10^6^ ha (ref. ^21^)). This equates to 1.1% of Indonesia’s most recently reported total GHG emissions in 2019 (ref. ^49^). Infrequent but intense fires are common in unmanaged degraded peatlands, particularly during prolonged drought driven by climate extreme events (for example, 2006, 2015 and 2019), and may result in higher GHG release to the atmosphere than peat decomposition^49^. GHG emissions at the degraded site are about 20% higher than those of the plantation site, indicating that establishment of *Acacia* plantation on previously degraded site could apparently result in lower long-term GHG emissions of −7.5 ± 6.5 tCO_2_-eq ha^−1^ year^−1^, as well as avoided emissions from bioenergy production (Fig. 2). Because the initial disturbance of this site occurred at a similar time to that at the plantation site (see Methods), it is probable that higher emissions from the degraded site are partly because of the decomposition of woody debris from fallen dead trees. Our results do not argue against full restoration of unmanaged degraded peatlands where this is achievable, as their ecosystem rehabilitation (that is, hydrological restoration and re-establishment of a closed forest canopy) offers an opportunity to restore and improve the ability of peatlands to sequester and retain C, but this will be critically dependent on protecting these areas from encroachment and fire.

Using our EFs from the intact and degraded sites, the results highlight that, despite evidence that they may now be losing C, avoided emissions from conserving all remaining intact peat swamp forests in Indonesia (2.0 × 10^6^ ha) under Indonesia’s nationally determined contribution^49^ and emissions reduction from restoring 4.2 × 10^6^ ha by 2050 under Indonesia’s Low Carbon scenario Compatible with the Paris Agreement target (LCCP)^50^ will avoid GHG emissions of around 160 MtCO_2_-eq year^−1^. This equates to around 40% of GHG emissions from peat decomposition in Indonesia in 2019 (ref. ^49^). This estimate is conservative. If some remaining intact peatlands are continuing to sequester CO_2_, the avoided emissions will be correspondingly higher. Our results clearly indicate that the net avoidance and reduction of GHG emissions resulting from peatland conservation, restoration and sustainable management represent a notable contribution to nationally determined contributions to a 1.5 °C world^32^.

## Methods

### Study area

This study was conducted in the Kampar Peninsula (Sumatra, Indonesia), an ombrogenous tropical peatland of around 700,000 ha that largely formed within the past 5,100 years (ref. ^8^). The base of the peatland is grey marine clays, over which peat varies from approximately 3 m deep near the river boundaries to over 11 m in the centre of the approximately 60-km-wide and more than 100-km-long peat dome (Fig. 1), with an average depth of 8 m. The peninsula experiences a humid tropical climate with the average monthly air temperature ranging from 26 to 29 °C (refs. ^17,27^). The variability in rainfall is influenced by monsoonal processes combined with El Niño–Southern Oscillation and IOD^51,52^. In general, the El Niño^34^ and positive IOD^35^ occur sequentially, with the positive IOD peaking a few months after the El Niño, exerting a strong combined effect on regional rainfall patterns^26^. The average annual rainfall for the past 8 years (2014–2021, with El Niño in 2015, La Niña in 2017 and a major positive IOD combined with an El Niño event in 2019) is 1,772 ± 201 mm. Rainfall varies seasonally, with two annual peaks, one in November–December and another in March–April. The land cover of the peninsula is characterized by a large central forest area that still has good-quality dense forest, representing one of the largest peat swamp forests in Southeast Asia. In some parts of the peninsula, selective logging took place in the 1990s, including the construction of access logging tracks and canals, especially around the periphery of the forest. However, some areas have never been logged and have been classified as intact peat swamp forest^21^. Most of the logged forest was converted to industrial fibre wood plantation and smallholder agriculture in the early 2000s. At present, the central forest area is surrounded by a mosaic of *A. crassicarpa*, oil palm plantation and degraded peat swamp forest with shrub and open land^21^ (Fig. 1).

At the experimental fibre wood plantation site, the peat swamp forest was disturbed by selective logging activity, including logging tracks and canals in the early 1990s. In the early 2000s, the area was converted to an *Acacia* plantation. This involved clearance of the remaining logged forest, artificial compaction during mechanical land preparation, installation of regularly spaced water management and access canals and planting of *A. crassicarpa*, which is harvested on a 4–5-year rotation. The area was not affected by fire before, during or after land-use change. *Acacia crassicarpa* (Leguminosae) is a fast-growing, N-fixing tree that is the principal fibre wood plantation species grown on peat soils in Southeast Asia. The typical plantation rotation period between planting of tree seedlings to harvest is 4–5 years, and a closed canopy develops in around 12 to 18 months. When measurements began in October 2016, the trees were already at the end of the third plantation rotation. All plantation compartments within a 2-km radius around the eddy covariance tower were harvested between October 2016 and April 2017. Tree height at harvest was in the range 19–24 m, determined from a vegetation survey in permanent sampling plots (20 m × 125 m) around the tower. Replanting for the fourth plantation rotation took place within two weeks after harvesting each individual compartment at a density of 1,667 trees per hectare (3 m × 2 m spacing). Five grams of chelated micronutrients per tree were applied around the seedlings during planting. All compartments within a 2-km radius of the eddy covariance tower were harvested between June and August 2021, when the average plantation age was 4.7 years, and replanting for the fifth plantation rotation took place within two weeks after harvesting. The ground surface in the plantation area is relatively even, without a hummock-hollow microtopography and with very little understory vegetation. The site soil characteristics are summarized in Extended Data Table 1. GWLs in the experimental plantation are actively managed by means of an extensive network of topographically defined water management zones, controlled by outlet sluices and supported by GWL monitoring. Water management zones consist of navigable canals, typically of 12 m width and 3 m depth, also used for transport^25^. Branch canals of 5–8 m width run perpendicular to these canals at a spacing of 500–800 m to form plantation compartments, which contain 1-m-deep field drains at a spacing of 75 m (ref. ^25^). An integrated climatologic footprint analysis^53^ indicated that (1) approximately 80% of measured fluxes derived from within 1,000 m in the upwind direction and thus originated within the *Acacia* plantation and (2) the water surface of ditches and canals represented 2% of the flux footprint (Fig. 1).

The second eddy covariance tower is located on the boundary of the degraded peatland and *Acacia* plantation (Fig. 1). To represent only the degraded peatland, half-hourly measurements in which the prevailing wind came from the plantation site (90° to 270°) were excluded, as is commonly done in eddy covariance studies^54^. The degraded site was selectively logged and drained in the late 1990s and early 2000s, whereas some parts were burned in 2014. The average canopy height was about 19 m. The tree density with a diameter at breast height of greater than 5 cm was 663 trees per hectare. Some large trees had been logged or fallen and many of those remaining were leaning. The site characteristics are summarized in Extended Data Table 1. The integrated climatologic footprint analysis^53^ indicated that approximately 80% of the fluxes were derived within 1,000 m in the upwind direction and the previously burnt area represented around 5% of the flux footprint (Fig. 1). The average eddy covariance footprint can be considered typical of many unmanaged degraded peatlands in Southeast Asia^21^.

The intact peat swamp forest structure is mixed with an uneven canopy (average canopy height = 32 m). The density of trees with a diameter at breast height greater than 5 cm was 1,343 stems per hectare. The vegetation and soil characteristics are summarized in Extended Data Table 1. The GWL fluctuates following the rainfall variation because of the ombrotrophic nature of the area^17,27^. An integrated climatologic footprint analysis^53^ indicated that approximately 80% of the fluxes were derived within 1,000 m in the upwind direction (Fig. 1) and thus originated from intact forest with neither logging nor canal-construction activity^21^. Some long-term regional effects of hydrological management of surrounding plantations cannot be ruled out, but a previous analysis suggested that the strongest effects occurred within 300 m of the plantation boundary^25^, and recent multivariate analysis indicates that subsidence in the interior forest is independent of distance from plantation canals^26^. The nearest active plantation is 3.5 km from the flux tower and well outside the flux footprint. Further, to avoid any possible boundary effect and associated bias, measurements from a wind direction between 78° and 191° were excluded in this study (Fig. 1).

Eddy covariance provides half-hourly measurements of turbulent exchanges between an entire ecosystem and the atmosphere above the vegetation canopy^54^. Hence, eddy covariance measurements incorporate all existing sources and uptakes that can vary substantially within an ecosystem in both space and time arising from variation in environmental conditions. Given the flat terrain (slope less than 0.05%), using the measured vegetation-canopy height and wind speed, the estimated 80% eddy covariance flux footprints represent an area of interest of around 1,000 m radius (Fig. 1). Flux measurements with the eddy covariance technique are expensive and high maintenance, and few studies include replicated measurements from several towers in tropical forested ecosystems. The relatively close proximity of the *Acacia* plantation and intact and degraded sites (Fig. 1) within the same peat landscape avoids potentially confounding variables such as differences in past natural succession^55^ and peat formation^8^.

### Eddy covariance and environmental variable measurements

Each eddy covariance system consisted of an enclosed-path CO_2_/H_2_O analyser (LI-7200, LI-COR) to measure CO_2_ and H_2_O concentrations, an open-path CH_4_ analyser (LI-7700, LI-COR) to measure CH_4_ concentrations and a three-dimensional sonic anemometer (WindMaster Pro 3-Axis Anemometer, Gill Instruments) to measure the orthogonal components of wind-speed fluctuations. Eddy covariance sensors were mounted at the top of each tower to ensure complete exposure in all directions (Fig. 1). The filters of the CO_2_ analyser were manually cleaned, either biweekly or if the flow drive increased above 80% (indicating filter clogging). The mirrors of the CH_4_ analyser were cleaned automatically either at 05:00 local time every day or if the received-signal-strength indicator dropped below 20%, because CH_4_ data become noisy below this threshold. Furthermore, the upper and lower mirrors of the CH_4_ analyser were manually cleaned on a biweekly basis. The raw eddy covariance turbulence data were recorded at 10 Hz using an analyser interface unit (LI-7550, LI-COR) and were stored on a removable flash disk (Industrial Grade USB Flash Disk, APRO).

A quantum sensor (LI-190SL-50, LI-COR) was mounted at the top of each tower to measure incoming photosynthetic photon flux density (PPFD). A radiometer (CNR4, Kipp & Zonen) was also mounted at the top of each tower to measure global and net radiation. Vertical profiles of relative humidity and air temperature were measured using air-temperature and humidity probes (HMP155, Vaisala), which were installed inside ventilated radiation shields at five heights from the ground surface, 3, 7, 13, 23 and 40 m for the plantation site, 3, 7, 14, 21 and 40 m for the degraded site and 4, 11, 20, 29 and 48 m for the intact site. Vertical profiles of the CO_2_ concentrations were measured by air sampling at four heights, 3, 12, 22 and 40 m for the plantation site, 3, 14, 21 and 40 m for the degraded site and, 4, 11, 29 and 48 m for the intact site, to calculate the flux-storage^56^ term below the measurement height using a closed-path CO_2_ analyser (LI-8100, LI-COR). The air-sampling intakes were automatically changed every 90 s and the CO_2_ concentration was measured for the last 10 s of every 90-s sampling time at each sampling height and recorded using a data logger (LI-8100, LI-COR); therefore, one rotation of measurements took 6 min in every 30 min. Both the enclosed-path and closed-path CO_2_ analysers were calibrated every three months using reference gases with concentrations of 396 and 444 ppm CO_2_ in air (certified grade ±1 ppm) and ultrahigh-purity nitrogen as the zero-point gas. The soil temperature was measured at 0.15 m below the hollow peat surface using a temperature probe (HydraProbe II, Stevens Water Monitoring Systems) from September 2017 until November 2018 and from October 2016 until June 2020 with three replicates at the intact and plantation sites, respectively. From November 2019 until May 2022, the soil temperatures were measured at the intact site and from November 2019 until May 2021 at the plantation site with two replicates using a temperature probe (AquaCheck, South Africa). The soil temperatures were not measured at the degraded site owing to site logistic issues.

All meteorological sensors took measurements every second and were recorded as 1-min averages using a data logger (Model 9210 XLite, Sutron). Each measuring system was powered using five solar panels (65-W solar panel, SunWize), along with eight rechargeable batteries (6 V and 305 Ah, Sun Xtender). The daily rainfall (mm day^−1^) was manually measured using three, two and three bucket-rain gauges within a distance of 11 km from the tower location at the plantation, degraded and intact sites, respectively. Each rain gauge was installed 1.5 m above the ground, in an open area so that rainfall was not affected by surrounding vegetation.

The GWL was monitored as the water elevation relative to the ground surface, taking the base of the hollows as a datum^17^. Data were recorded as negative distance below the surface, with positive values indicating ponding above the surface. The GWL loggers (four around the plantation tower, one in the degraded site and six in the intact site) to record the GWL every 30 min using a pressure transducer (Levelogger Model 3001, Solinst) were placed in perforated polyvinyl chloride pipes that were inserted vertically into the peat and anchored into the underlying clay (Fig. 1). Each GWL logger also recorded the water temperature in the pipe 1.5 m below the peat surface. Further GWL data were manually recorded biweekly at seven and three locations at the plantation and degraded sites, respectively, and on a quarterly basis at eight more locations in the intact site (Fig. 1).

Peat subsidence was measured at 11, four and 14 locations in the plantation, degraded and intact sites, respectively (Fig. 1), with hollow, perforated 5-cm-diameter polyvinyl chloride poles inserted vertically into the peat and anchored into the underlying mineral subsoil following the approach described in ref. ^25^. Annual average subsidence rates were derived from measurements during October 2016–May 2021 for the plantation site and during December 2017–May 2022 for the degraded and intact sites.

For peat physical and chemical properties of the surface layer (0–50 cm), four plots in each of the *Acacia* plantation and degraded sites and three plots in the intact site were randomly selected within the eddy covariance flux footprint (200–1,000 m distance from each tower location; Fig. 1). At each plot, ten subsamples within a 200-m radius were composited. Peat samples for bulk density, pH and ash content were collected in September 2017, February 2019 and September 2019 in the intact site, June 2017, January 2018, October 2018 and February 2019 in the degraded site and June 2017, February 2018, October 2018, February 2019 and October 2019 in the *Acacia* plantation. Samples for soil C, N, nitrate and ammonium content were collected in August 2020 and October 2021 for all sites.

For the *Acacia* plantation site, time‐integrated NEE-CO_2_ over the plantation rotation was combined with C export in the harvested wood. Total C export in harvested wood and delivered to the mill from the total footprint area of 220 ha over the average plantation age of 4.7 years was calculated using a basic density of 455 ± 25 kg m^−3^ and average C content of 48.2% (refs. ^56–59^). The exported wood is converted into pulp products and biomass fuel for bioenergy generation. We applied the conservative assumption that all C in exported wood would be returned to the atmosphere as CO_2_. Intact and degraded sites were considered to have had no biomass C export during the study period.

The biomass C loss owing to land-use change was calculated from aboveground and belowground biomass C-stock differences between the intact site and the *Acacia* plantation and degraded areas. Aboveground and belowground biomass were determined using seven permanent sampling plots (20 m × 125 m) at each site and following the allometric equations described in refs. ^60,61^ for the intact and degraded sites and ref. ^62^ for the *Acacia* plantation. A time horizon of 100 years of land being used after conversion is chosen on the basis of ISO 14067 on C footprint.

### Eddy covariance data processing

The eddy covariance fluxes of CO_2_, CH_4_ and evapotranspiration were computed from the 10-Hz concentration and vertical wind velocity data using EddyPro software (version 6.2.0, LI-COR) at a standard half-hour averaging interval^54^. A despiking procedure was applied to detect and eliminate individual out-of-range values for vertical wind velocity and concentration data^63^. Detrending was carried out using the block-averaging method. A coordinate correction was applied to force the average vertical wind velocity to zero by the planar-fit method^64^. Frequency response loss corrections were applied to compensate for the flux losses at low and high frequencies^65^. The Webb–Pearman–Leuning correction^66^ for air-density fluctuations induced by temperature (thermal expansion) and water vapour (dilution) was applied.

Differences between deployment-specific variables, that is, the sensor separation distance and instrument placement, were considered when processing the data. The half-hourly CO_2_ storage below the flux measurement height was calculated from the four-point vertical profiles of CO_2_ concentration, relative humidity and air temperature by temporal interpolation^56^. Finally, the net ecosystem CO_2_ exchange was calculated as the sum of the storage flux and the eddy covariance flux. Owing to the large power requirement and cost of a separate CH_4_ analyser, we could not conduct CH_4_-profile measurements to calculate the CH_4_ storage^67^. In theory, accumulated CH_4_ below the canopy during the nighttime is probably released and measured by the eddy covariance system following the onset of turbulence after sunrise, and the bias on annual sums should be negligible^67^.

After a set of quality controls^68–70^ and system malfunctions and power-supply failure mainly because of lightning strikes, the numbers of high-quality measurements during the course of the study were 37%, 34% and 34% for CO_2_, 26%, 29% and 25% for CH_4_ and 34%, 34% and 28% for evapotranspiration in the plantation, degraded and intact sites, respectively. A similar range of 25–50% has been reported for other eddy covariance studies in tropical forested peatlands^42,56^. The remaining half-hourly measurements that met all the quality criteria totalled 30,196, 14,330 and 18,136 for CO_2_, 21,305, 12,721 and 13,026 for CH_4_ and 27,965, 14,437 and 14,919 for evapotranspiration for the plantation, degraded (270–90° wind direction) and intact (191–78° wind direction) sites, respectively.

We gap-filled both low-quality and missing data, as is commonly done in eddy covariance studies^17,27,42,56,71–75^. Following ref. ^17^, we applied three gap-filling approaches for CO_2_: (1) marginal distribution sampling (MDS)^56,76^, (2) artificial neural network (ANN)^73^ and (3) random forest (RF)^74^ separately for the daytime (06:00–18:00 local time) and the nighttime (18:00–06:00 local time) data. To avoid any possible gap-filling bias in estimates of CO_2_, CH_4_ and evapotranspiration, we used the average of the three approaches^17^. We applied principal component analysis as an input to the algorithms to address multidriver dependency of CO_2_ exchange and reduce the internal complexity of the algorithmic structures for the MDS approach^77^, using PPFD, vapour-pressure deficit (VPD) and air temperature during daytime. Nighttime CO_2_ exchanges were considered equivalent to the ecosystem respiration (*R*_eco_) value^78^. The GWL is reported as the main controlling factor of *R*_eco_ from tropical peatlands^17,56^. Therefore, we used the GWL, air temperature and soil temperature as environmental factors for the lookup table to derive the nighttime CO_2_ exchanges using the MDS gap-filling algorithm. Following other regional eddy covariance studies in peat swamp forests^17,56^, we performed MDS gap-filling using the REddyProc package (https://CRAN.R-project.org/package=REddyProc) on a half-hourly basis^77^. ANN and RF procedures were iterated 20 times. For ANN and RF, PPFD, VPD, air temperature, GWL and friction velocity were used as predictive variables for the daytime and the PPFD and VPD data were excluded in the nighttime.

We applied the above gap-filling approaches for CH_4_ and evapotranspiration as well. For CH_4_, we used GWL, VPD, air temperature, friction velocity, latent heat flux, sensible heat flux, atmospheric pressure and global radiation in the daytime and latent heat flux and global radiation were excluded in the nighttime for ANN and RF. For MDS, we used latent heat flux, GWL and air temperature during the daytime and GWL, air temperature and soil temperature during the nighttime. For evapotranspiration, we applied net radiation instead of PPFD during the daytime, whereas net radiation and VPD were excluded during the nighttime. After gap filling, we corrected the daily evapotranspiration for the energy imbalance using net radiation, sensible heat and latent heat as described in ref. ^79^.

The flux random uncertainty was calculated following ref. ^80^. The standard deviation of three different flux values derived from friction velocity thresholds of the 5th, 50th and 95th percentiles were applied as an uncertainty because of the friction velocity threshold using the REddyProc package^77^. The gap-filling flux uncertainty was calculated from the standard deviation of the MDS procedure^77^. Averages of the 20 ANN and RF modelled values were used to fill gaps and the standard deviation was used to quantify the uncertainty owing to gap filling. The total uncertainty in eddy covariance measurements of CO_2_, CH_4_ and evapotranspiration included gap-filling, random and friction velocity uncertainty^81^. The annual estimate of CO_2_, CH_4_, evapotranspiration and GHG balance includes total uncertainty calculated using the propagation of errors law.

### Reporting summary

Further information on research design is available in the Nature Portfolio Reporting Summary linked to this article.

## Online content

Any methods, additional references, Nature Portfolio reporting summaries, source data, extended data, supplementary information, acknowledgements, peer review information; details of author contributions and competing interests; and statements of data and code availability are available at 10.1038/s41586-023-05860-9.

### Supplementary information

Supplementary Information Supplementary Methods 1–4 and Supplementary References.

Reporting Summary

Peer Review File

## Figures and Tables

**Extended Data Table 1:**
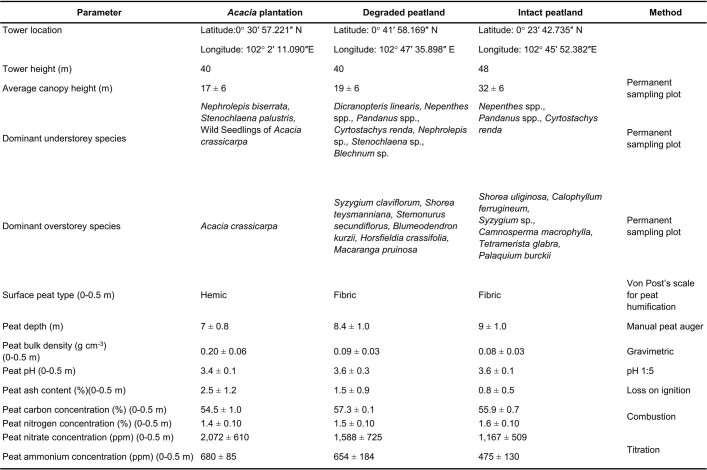
Characteristics of *Acacia* plantation and degraded and intact peat swamp forest in Sumatra, Indonesia

Values represent averages with standard deviation.

**Extended Data Table 2:**
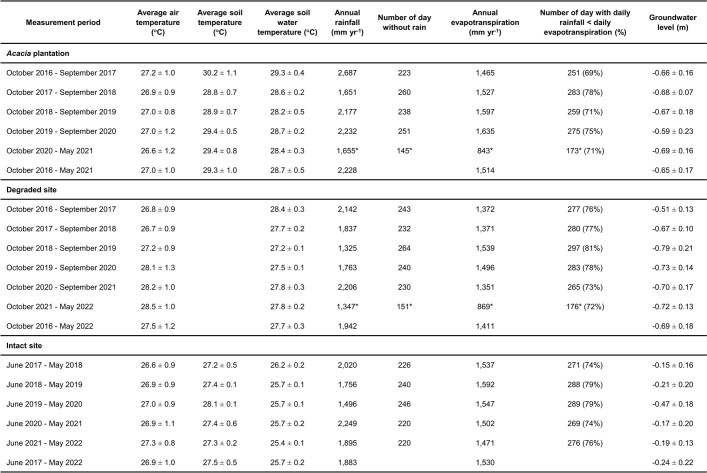
Annual average with standard deviation of environmental variables at *Acacia* plantation and degraded and intact peat swamp forest in Sumatra, Indonesia

GWLs were averaged from 11 locations around the *Acacia* plantation tower site, four locations around the degraded tower site and 15 locations in the intact site. The numbers marked with an asterisk (*) are cumulative estimates over eight months. Negative GWL values indicate the water level below the peat hollow surface.

**Extended Data Table 3:**
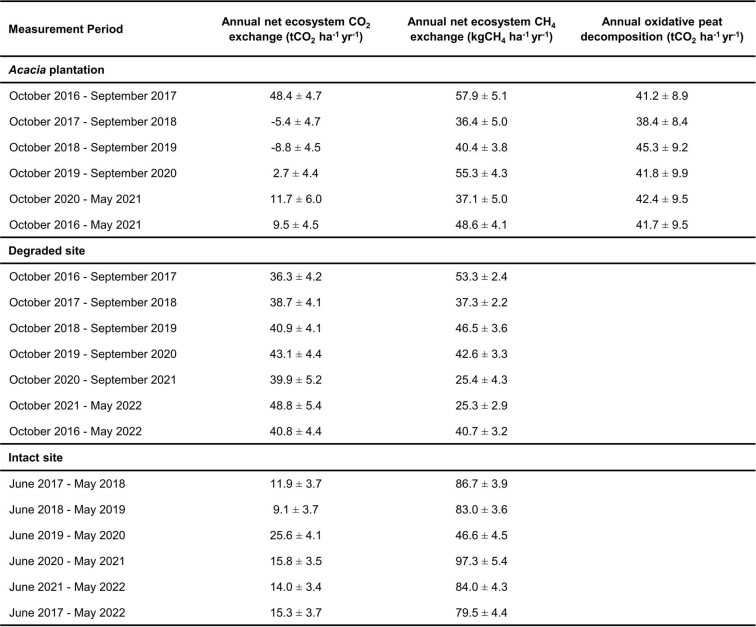
Net ecosystem CO_2_ and CH_4_ exchanges measured using eddy covariance over *Acacia* plantation and degraded and intact peat swamp forest in Sumatra, Indonesia

Annual estimates with total uncertainty includes random error, friction velocity criteria and gap-filling approach. Annual oxidative peat decomposition measured using the soil-chamber technique at the *Acacia* plantation is given with standard deviation from four soil flux chambers. Positive values indicate emission to the atmosphere, negative values indicate net uptake by the ecosystem.

**Extended Data Table 4:**
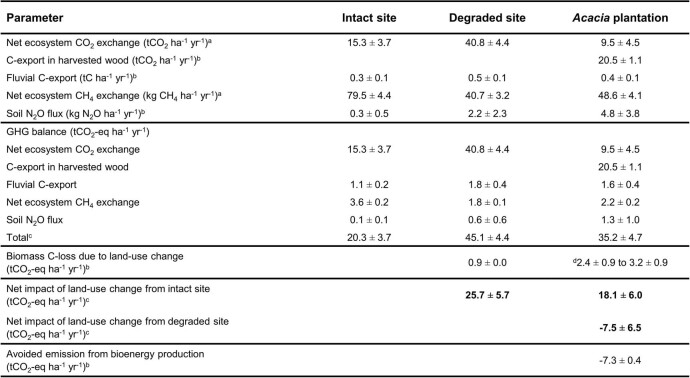
GHG balance of *Acacia* plantation and degraded and intact peat swamp forest in Sumatra, Indonesia

To quantify total GHG balance in CO_2_ equivalent (CO_2_-eq), we used a sustained-flux global-warming potential (SGWP) of 1, 45 and 270 for CO_2_, CH_4_ and N_2_O, respectively, over a 100-year time period^39^. Total GHG balance = (net ecosystem CO_2_ exchange + net ecosystem CH_4_-C exchange + fluvial C export + C export in harvested wood, where applicable) + (net ecosystem CH_4_ exchange × SGWP) + (soil N_2_O flux × SGWP). We assumed that all fluvial C export is ultimately converted to CO_2_ (ref. ^38^). Avoided emissions from bioenergy production is calculated by assuming that 54% of harvested wood is used for bioenergy production (details in Supplementary Method 4). ^a^Values represent average with total uncertainty from random error, friction velocity threshold and gap-filling approach. ^b^Values represent average with standard deviation. ^c^Values represent total with standard deviation calculated from propagation of errors. ^d^Lower and upper ranges represent biomass C loss owing to establishment of *Acacia* plantation on the degraded and intact sites, respectively. The bold numbers indicate net impact of land-use change. Positive value indicates emission to the atmosphere and negative value indicates avoided emission.

**Extended Data Table 5:**
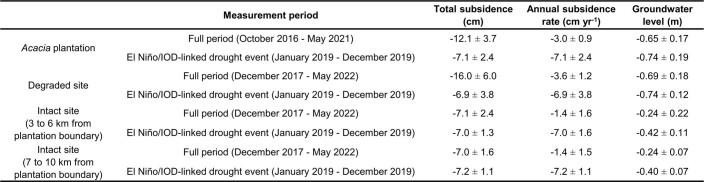
Average and standard deviation of peat subsidence at *Acacia* plantation and degraded and intact peat swamp forest in Sumatra, Indonesia

Subsidence rates are strongly affected by the El Niño/IOD-linked drought event in 2019. Peat subsidence rates were derived from 11 locations in the *Acacia* plantation, four locations in the degraded site and 14 locations in the intact site (see Methods). Negative peat subsidence values indicate that the ground surface was falling. Negative GWL values indicate the water level below the peat hollow surface.

**Extended Data Table 6:**
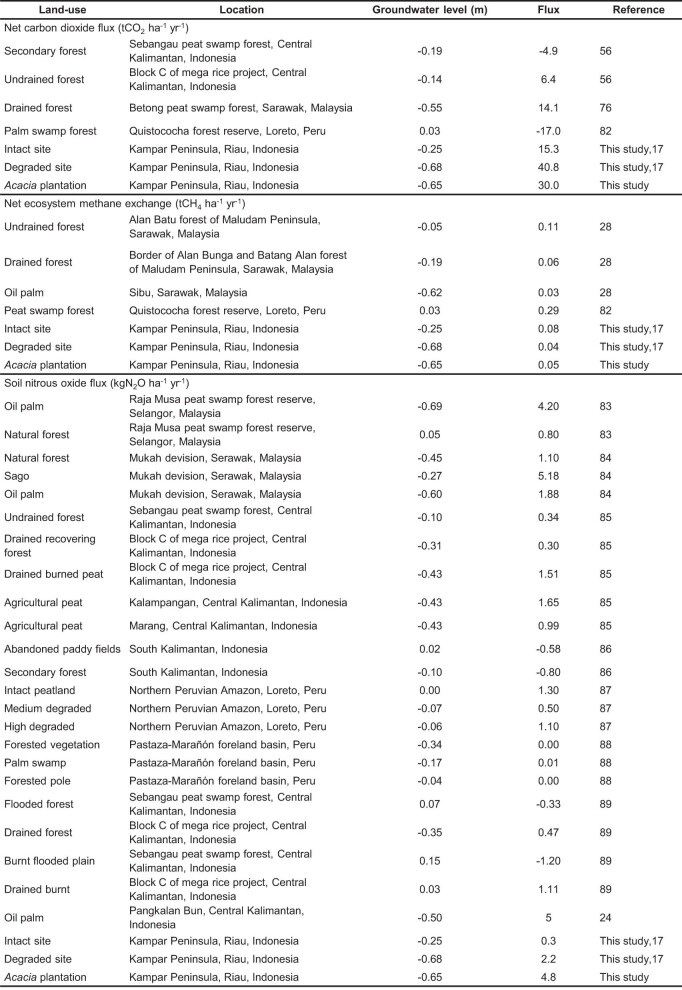
Land use and locations of tropical peatland sites used in net ecosystem exchanges of CO_2_ and CH_4_ and soil N_2_O syntheses

Positive and negative GWL values indicate the water level above and below the peat hollow surface, respectively. Positive flux value indicates emission to the atmosphere and negative value indicates uptake by the ecosystem. References ^82–90^.

## Data Availability

All data that support the findings of this study are archived on Zenodo at 10.5281/zenodo.7728463.
