# Supplementary material for: Net greenhouse gas balance of fibre wood plantation on peat in Indonesia
Source: Nature. 2023 Apr 5;616(7958):740–6. doi: 10.1038/s41586-023-05860-9 (PMC10132972; doi:10.1038/s41586-023-05860-9)
Supplement: Supplementary file 1 — Supplementary Methods 1–4 and Supplementary References. [file 41586_2023_5860_MOESM1_ESM.pdf]

nature portfolio

https://doi.org/10.1038/s41586-023-05860-9

Supplementary information

Net greenhouse gas balance of fibre wood plantation on peat in Indonesia

In the format provided by the authors and unedited

Supplementary Method

Title: Net greenhouse gas balance of fiber wood plantation on peat in Indonesia

Author list and affiliations

Chandra S. Deshmukh[1,*], Ari P. Susanto[1], Nardi[1], Nurholis[1], Sofyan Kurnianto[1], Yogi Suardiwerianto[1], M. Hendrizal[1], Ade Rhinaldy[1], Reyzaldi E. Mahfiz[1], Ankur R. Desai[2], Susan E. Page[3], Alexander R. Cobb[4], Takashi Hirano[5], Frédéric Guérin[6], Dominique Serça[7], Yves T. Prairie[8], Fahmuddin Agus[9], Dwi Astiani[10], Supiandi Sabiham[11], Chris D. Evans[12]

[1]Asia Pacific Resources International Ltd, Pelalawan Regency, Indonesia
[2]Department of Atmospheric and Oceanic Sciences, University of Wisconsin-Madison, Madison, WI, USA
[3]School of Geography, Geology and the Environment, University of Leicester, Leicester, UK
[4]Singapore-MIT Alliance for Research and Technology, Singapore
[5]Research Faculty of Agriculture, Hokkaido University, Sapporo, Japan
[6]Geosciences Environnement Toulouse, CNRS, IRD, Université Paul-Sabatier, Toulouse, France
[7]LAERO, Université de Toulouse, CNRS, IRD, UT3, Toulouse, France
[8]UNESCO Chair in Global Environmental Change, Universite du Quebec a Montreal, Montreal, Quebec, Canada
[9]Indonesian Center for Agricultural Land Resources Research and Development, Bogor, Indonesia
[10]Faculty of Forestry, Tanjungpura University, Pontianak, Indonesia
[11]Department of Soil Science and Land Resource, IPB University, Bogor, Indonesia
[12]UK Centre for Ecology and Hydrology, Bangor, LL57 2UW, UK

*Corresponding author: Chandra S. Deshmukh chandra_deshmukh@aprilasia.com

Supplementary Method 1. Data collation for CO_2 and CH_4 fluxes

To place our results into a regional context, we collated a data set of published eddy covariance CO_2[1-3] and CH_4[3,4] flux measurements from tropical peatlands (*Extended Data Table 6*). We restricted the analysis to EC studies, on the basis that these provide spatially integrated data sets with high temporal resolution; this also ensured methodological consistency with the sites in our analysis. For CO_2 flux, oil palm sites[5,6] were not included because no studies so far have reported C-balance over a full plantation rotation, and thus they are not in steady state with regard to CO_2 uptake and emission, making the underlying peat C-balance uncertain. However, naturally forested and degraded sites were considered to have had no biomass C-export during the study period.

Supplementary Method 2. Soil N_2O flux measurements

Soil N_2O fluxes were measured using manual flux chamber technique[7]. Four plots in each of the *Acacia* plantation and degraded site, and three plots in the intact were randomly selected within EC flux footprint (200 - 1,000 m distance from the EC tower location *Fig. 1*). At each plot in the degraded and intact sites, two stainless steel rectangular collars (surface area = 0.08 m^2) on hummocks and four in the adjacent hollows, whereas four collars per plot in the plantation (randomly around 50-100 m apart) were inserted permanently 15 cm into the peat five months before the start of the flux monitoring. A groove in the top of the collar was filled with mineral water before connection of the chamber to provide an airtight seal. At each sampling time and at each collar, a stainless steel chamber (volume = 12 liter) was placed onto the collar. Within 45 min, four air samples were collected from the chamber using a syringe and

needle at 15 min intervals starting from the initial time when the chamber was placed onto the collar. The air within the chambers was gently mixed prior to sample collection using the syringe and needle. Air samples were transferred into pre-evacuated 20 ml glass vials capped with butyl rubber stoppers and aluminum seals. Analysis of N_2O concentrations was performed within 48 h by gas chromatography (8610C gas chromatograph, SRI Instruments) using an electron capture detector. Commercial gas standard with concentrations of 350 and 550 ppb (parts per 10^9) N_2O in N_2 (with an uncertainty of less than 10%) was injected for calibration after the analysis of every ten samples. The N_2O fluxes were calculated applying the ideal gas law to the slope of the linear regression of the gas concentration in the chamber versus time. A total 308, 337 and 328 flux measurements were made between December 2019 and March 2022 for the plantation site, between July 2019 and March 2022 for the degraded site, and between June 2019 and February 2022 for the intact site on a bi-monthly basis, covering the dry and wet seasons to represent the variation in the GWL and soil temperature. There was no significant difference ($p > 0.05$, Kruskal-Wallis test) between the N_2O fluxes at hollows and hummocks (*Extended Data Fig. 3*). We averaged the soil N_2O fluxes of all replicates from each sampling month, then averaged all monthly values to estimate average N_2O emissions. Thus, our estimates cover both spatial and seasonal variability. We also gathered a data set of published[8-14] N_2O flux data obtained soil chamber technique from tropical peatlands (*Extended Data Table 6*) and derived a relationship between N_2O fluxes and groundwater levels (*Extended Data Fig. 3*). We did not measure the emissions from tree stems, the inclusion of which may increase the

total annual N_2O emissions by 11-38%[15], although this would have little effect on total GHG balance due to the minor contribution of N_2O (<1%).

Supplementary Method 3. Oxidative peat decomposition measurements

At the *Acacia* plantation in May 2016, four rectangular stainless steel frame (width = 0.6 m, length = 0.7 m), ~10 - 15 m apart from each other, were inserted 1 m deep to prevent autotrophic respiration by roots in order to ensure that the measured CO_2 flux only represented the oxidative decomposition of peat[16]. Polyvinyl chloride (PVC) collars were inserted ~0.1 m deep in the soil inside the frame. In October 2016, an automated chamber system (LI-8100-104, LI-COR) consisting of four white enamel-coated stainless steel chambers (volume = 4,076 cm^3; surface area = 320 cm^2) was installed and connected with the same analyzer used for vertical profiles of the CO_2 concentrations. Each chamber was closed for 140 s (15 s pre-purge, 20 s dead-band, 90 s measurements and 15 s post-purge) in sequence, one after the other, and it took 9 min for all four chambers to close/open in rotation. The air in the headspace of each chamber was circulated through the CO_2 analyzer when the chambers were closed. The CO_2 concentration was measured at 1 s intervals and recorded in the data logger. We removed measurements with negative fluxes, coefficients of regression of <0.9, or values that were extreme outliers (≥99th percentile). After quality control and instrument malfunction due to lightning strike, 128,615 (39.3% of the total) half-hourly measurements remained available. To capture diel pattern to avoid any possible bias [17,18], the daily mean oxidative peat decomposition was calculated only when the number of available data points was larger than six in both the daytime (6:00-18:00 local time) and nighttime (18:00-6:00 local time), respectively. Following ref[18], a relationship

between available daily oxidative peat decomposition and corresponding GWL was derived from the quality controlled data and used to calculate daily oxidative peat decomposition using GWL for the missing days. We combined oxidative peat decomposition with the NEE-CO_2 and C-export in harvested wood to quantify a conservative estimate of C-input to the peat from litter, roots, stumps and bark residues over a full plantation rotation.

Supplementary Method 4. Calculation of avoided emission from bioenergy

A one-hectare plantation on peat delivers 11.6 ± 0.6 tonnes of dry wood annually to the pulp mill. In addition, a 0.46 wood to pulp conversion factor[19], 5.3 tonnes of delivered dry wood becomes pulp and the remaining portion of dry wood produces 77 ± 4.0 GJ of bioenergy using a net calorific value of 12.3 GJ per tonne dry biomass[20], replacing coal combustion and associated emissions. Our estimated avoided emission was 7.3 ± 0.4 tCO_2-eq ha^{-1} yr^{-1}, calculated using an emission intensity per energy produced from coal combustion of 0.095 tCO_2-eq per GJ[21].

Supplementary references

1. Hirano, T. et al. Effects of disturbances on the carbon balance of tropical peat swamp forests. *Glob. Change Biol*. **18**, 3410-3422 (2012).
2. Kiew, F. et al. CO_2 balance of a secondary tropical peat swamp forest in Sarawak, Malaysia. *Agric. For. Meteorol*. **248**, 494-501 (2018).
3. Griffis, T. J. et al. Hydrometeorological sensitivities of net ecosystem carbon dioxide and methane exchange of an Amazonian palm swamp peatland. *Agric. For. Meteorol.* **295**, 108167 (2020).

4. Wong, G. X. et al. How do land use practices affect methane emissions from tropical peat ecosystems? *Agric For Meteorol*. 282-283:107869 (2020).
5. Kiew, F. et al. Carbon dioxide balance of an oil palm plantation established on tropical peat. *Agric. For. Meteorol*. **295**, 108189 (2020).
6. McCalmont J. et al. Short-and long-term carbon emissions from oil palm plantations converted from logged tropical peat swamp forest. *Glob. Change Biol.* **27**, 2361-2376 (2021).
7. Serça, D., Delmas, R., Jambert, C. & Labroue, L. Emissions of nitrogen oxides from equatorial rain forest in central Africa: origin and regulation of NO emission from soils. *Tellus B* **46,** 243-254 (1994).
8. Azizan, S. N. F. et al. Comparing GHG Emissions from Drained Oil Palm and Recovering Tropical Peatland Forests in Malaysia. *Wate*r **13**, 3372 (2021).
9. Melling, L., Hatano, R. & Goh, K. J. Nitrous oxide emissions from three ecosystems in tropical peatland of Sarawak, Malaysia. *Soil Sci. Plant Nutr*. **53**, 792-805 (2007).
10. Jauhiainen, J. et al. Nitrous oxide fluxes from tropical peat with different disturbance history and management. *Biogeosciences* **9**, 1337-1350, (2012).
11. Inubushi, K., Furukawa, Y., Hadi, A., Purnomo, E. & Tsuruta, H. Seasonal changes of CO_2, CH_4 and N_2O fluxes in relation to land-use change in tropical peatlands located in coastal area of South Kalimantan. *Chemosphere* **52,** 603-8 (2003).
12. Hergoualc'h, K. et al. Spatial and temporal variability of soil N_2O and CH_4 fluxes along a degradation gradient in a palm swamp peat forest in the Peruvian Amazon. *Glob. Change Biol*. **26,** 7198-7216 (2020).

13. Teh, Y. A., Murphy, W. A., Berrio, J., Boom, A. & Page, S. E. Seasonal variability in methane and nitrous oxide fluxes from tropical peatlands in the Western Amazon basin. *Biogeosciences* **14,** 3669-3683 (2017).
14. Adji, F, F., Hamada, Y., Darang, U., Limin, S. H. & Hatan, R. Effect of plant-mediated oxygen supply and drainage on greenhouse gas emission from a tropical peatland in Central Kalimantan. Indonesia. *Soil Sci. Plant Nutr*. **60**:2, 216-230 (2014).
15. Iddris, N. A. A., Corre, M. D., Yemefack, M., van Straaten, O. & Veldkamp, E. Stem and soil nitrous oxide fluxes from rainforest and cacao agroforest on highly weathered soils in the Congo Basin. *Biogeosciences* **17**, 5377-5397 (2020).
16. Itoh, M., Okimoto, Y., Hirano, T. & Kusin, K. Factors affecting oxidative peat decomposition due to land use in tropical peat swamp forests in Indonesia. *Sci. Total Environ*. **609**, 906-915 (2017).
17. Hoyt, A. M. et al. CO_2 emissions from an undrained tropical peatland: Interacting influences of temperature, shading and water table depth. *Glob. Change Biol*. **25**, 2885-2899 (2019).
18. Ishikura, K. et al. Soil carbon dioxide emissions due to oxidative peat decomposition in an oil palm plantation on tropical peat. *Agric. Ecosyst. Environ*. **254**, 202-212 (2018).
19. Suhr, M. et al. Best Available Techniques (BAT) Reference Document for the Production of Pulp, Paper and Board. Luxembourg: *Publications Office of the European Union* (2015).

20. Garg, A., Kazunari, K. & Pulles, T. Introduction. In Eggleston, H. S., Buendia, L., Miwa, K., Ngara, T. & Tanabe, K. (Eds.), 2006 IPCC Guidelines for National Greenhouse Gas Inventories: Energy. IGES, Japan (2006).
21. Gomez, D. R. et al. Stationary combustion. In Eggleston H. S., Buendia L., Miwa K., Ngara T. & Tanabe K. (Eds.), 2006 IPCC Guidelines for National Greenhouse Gas Inventories: Energy. IGES, Japan (2006).
