# Supplementary material for: Net greenhouse gas balance of fibre wood plantation on peat in Indonesia
Source: Nature. 2023 Apr 5;616(7958):740–6. doi: 10.1038/s41586-023-05860-9 (PMC10132972; doi:10.1038/s41586-023-05860-9)
Supplement: Supplementary file 2 — Reporting Summary [file 41586_2023_5860_MOESM2_ESM.pdf]

nature portfolio

Corresponding author(s): Chandra Shekhar Deshmukh

Last updated by author(s): Jan 3, 2023

Reporting Summary

Nature Portfolio wishes to improve the reproducibility of the work that we publish. This form provides structure for consistency and transparency in reporting. For further information on Nature Portfolio policies, see our Editorial Policies and the Editorial Policy Checklist.

Statistics

For all statistical analyses, confirm that the following items are present in the figure legend, table legend, main text, or Methods section.

n/a	Confirmed	
☐	☒	The exact sample size (*n*) for each experimental group/condition, given as a discrete number and unit of measurement
☐	☒	A statement on whether measurements were taken from distinct samples or whether the same sample was measured repeatedly
☐	☒	The statistical test(s) used AND whether they are one- or two-sided<br>*Only common tests should be described solely by name; describe more complex techniques in the Methods section.*
☒	☐	A description of all covariates tested
☐	☒	A description of any assumptions or corrections, such as tests of normality and adjustment for multiple comparisons
☐	☒	A full description of the statistical parameters including central tendency (e.g. means) or other basic estimates (e.g. regression coefficient) AND variation (e.g. standard deviation) or associated estimates of uncertainty (e.g. confidence intervals)
☐	☒	For null hypothesis testing, the test statistic (e.g. *F*, *t*, *r*) with confidence intervals, effect sizes, degrees of freedom and *P* value noted<br>*Give P values as exact values whenever suitable.*
☒	☐	For Bayesian analysis, information on the choice of priors and Markov chain Monte Carlo settings
☒	☐	For hierarchical and complex designs, identification of the appropriate level for tests and full reporting of outcomes
☒	☐	Estimates of effect sizes (e.g. Cohen's *d*, Pearson's *r*), indicating how they were calculated

Our web collection on statistics for biologists contains articles on many of the points above.

Software and code

Policy information about availability of computer code

Data collection	LI-7200RS commercial software (version 8.0.0, LI-COR) for downloading eddy covariance raw data;<br>LI-8100A commercial software (version 4.0.0, LI-COR) for downloading CO2 concentration profiles and oxidative peat decomposition data;<br>LoggerSW commercial software (version 4.0, Sollinst) for downloading the groundwater level data.
Data analysis	EddyPro commercial software (version 6.2.0, LI-COR) for preliminary CO2, CH4 and H2O eddy covariance data processing;<br>SoilFluxPro commercial software (version 4.0, LI-COR) for preliminary oxidative peat decomposition and storage term data processing.

For manuscripts utilizing custom algorithms or software that are central to the research but not yet described in published literature, software must be made available to editors and reviewers. We strongly encourage code deposition in a community repository (e.g. GitHub). See the Nature Portfolio guidelines for submitting code & software for further information.

Data

Policy information about availability of data

All manuscripts must include a data availability statement. This statement should provide the following information, where applicable:

- Accession codes, unique identifiers, or web links for publicly available datasets
- A description of any restrictions on data availability
- For clinical datasets or third party data, please ensure that the statement adheres to our policy

All data that support the findings of this study are archived on https://doi.org/10.5281/zenodo.7500659

Human research participants

Policy information about studies involving human research participants and Sex and Gender in Research.

Reporting on sex and gender	*Use the terms sex (biological attribute) and gender (shaped by social and cultural circumstances) carefully in order to avoid confusing both terms. Indicate if findings apply to only one sex or gender; describe whether sex and gender were considered in study design whether sex and/or gender was determined based on self-reporting or assigned and methods used. Provide in the source data disaggregated sex and gender data where this information has been collected, and consent has been obtained for sharing of individual-level data; provide overall numbers in this Reporting Summary. Please state if this information has not been collected. Report sex- and gender-based analyses where performed, justify reasons for lack of sex- and gender-based analysis.*
Population characteristics	*Describe the covariate-relevant population characteristics of the human research participants (e.g. age, genotypic information, past and current diagnosis and treatment categories). If you filled out the behavioural & social sciences study design questions and have nothing to add here, write "See above."*
Recruitment	*Describe how participants were recruited. Outline any potential self-selection bias or other biases that may be present and how these are likely to impact results.*
Ethics oversight	*Identify the organization(s) that approved the study protocol.*

Note that full information on the approval of the study protocol must also be provided in the manuscript.

Field-specific reporting

Please select the one below that is the best fit for your research. If you are not sure, read the appropriate sections before making your selection.

☐ Life sciences ☐ Behavioural & social sciences ☒ Ecological, evolutionary & environmental sciences

For a reference copy of the document with all sections, see nature.com/documents/nr-reporting-summary-flat.pdf

Ecological, evolutionary & environmental sciences study design

All studies must disclose on these points even when the disclosure is negative.

Study description	The study reports net ecosystem exchanges of carbon dioxide (CO2) and methane (CH4) as well as soil nitrous oxide (N2O) fluxes from Acacia crassicarpa plantation, degraded site and intact site within the same peat landscape to represent land-cover change trajectories in Sumatra, Indonesia. The study reports the first full plantation rotation GHG balance investigation undertaken in any fiber wood plantation on peatland globally. The GHG emissions from the Acacia plantation over a full plantation rotation were two times higher than those from the intact site, but only around half of the current IPCC Tier 1 emission factor for this land-use. Our results should help to reduce the uncertainty in the estimation of GHG emissions from globally important ecosystems, provide estimate of the impact of land-use change on tropical peat, and develop science-based peatland management practices as nature-based climate solutions that help to minimize GHG emissions.
Research sample	We quantified all major GHG flux terms (net ecosystem exchanges of CO2 and CH4, and soil N2O flux), including fluvial C-exports for Acacia crassicarpa plantation, degraded and intact sites within the same peat landscape in Sumatra, Indonesia. In addition, biomass C-loss due to plantation establishment and C-export in harvested wood for Acacia plantation were also quantified. The peat chemical and physical properties, groundwater level, and meteorological variables at all sites were measured.
Sampling strategy	We quantified GHG balance of fiber wood plantation on tropical peatland in Sumatra, Indonesia to cover a full plantation rotation (planting - plantation growth - harvesting) and all major GHG flux terms (net ecosystem exchanges of CO2 and CH4, and soil N2O flux), including biomass C-loss due to plantation establishment, C-export in harvested wood and fluvial C-exports. We compared the GHG balance at the fiber wood plantation with more than five years of measurements at the degraded and five years of measurements at the intact sites on the same peat landscape.
Data collection	Measurements of net ecosystem CO2 and CH4 exchanges were conducted using eddy covariance technique. Eddy covariance system consisted of an enclosed-path CO2/H2O analyzer (LI-7200, LI-COR) to measure CO2 and H2O concentrations, an open path CH4 analyzer (LI-7700, LI-COR) to measure CH4 concentrations, and a three-dimensional sonic anemometer (WindMaster Pro3-Axis Anemometer, Gill Instruments) to measure the orthogonal components of wind-speed fluctuations at 40, 40, and 48 m heights above ground surface for Acacia plantation, degraded, and intact sites, respectively. Soil N2O flux was measured using manual flux chamber technique, following gas chromatograph analysis. Peat oxidative decomposition was measured using automated chamber system (LI-8100-104, LI-COR) consisting of white enamel-coated stainless steel chambers connected to soil CO2 analyzer (LI-8100, LI-COR). For peat physical and chemical properties of the surface layer (0 - 50 cm) , four plots in each of the Acacia plantation and degraded site, and three plots in the intact site were randomly selected within the eddy covariance flux footprint (200 - 1,000 m distance from each tower location).
Timing and spatial scale	We collected continuous eddy covariance measurements over the full Acacia plantation cycle (planting - plantation growth - harvesting, October 2016 to May 2021), the degraded site (October 2016 to May 2022) and the intact site (June 2017 to May 2022). The raw eddy covariance data were recorded at 10 Hz frequency and fluxes were calculated at every 30 minutes. The eddy

covariance measurements represent an area within 1,000 m radius from the eddy covariance tower.
Soil N2O fluxes measurements were made between December 2019 and March 2022 for the plantation site, between July 2019 and March 2022 for the degraded site, and between June 2019 and February 2022 for the intact site on a bi-monthly basis. Four plots in each of the Acacia plantation and degraded site, and three plots in the intact within eddy covariance flux footprint (200 - 1,000 m distance from the eddy covariance tower location). At each plot in the degraded and intact sites, two stainless steel rectangular collars on hummocks and four in the adjacent hollows, whereas four collars per plot in the plantation (around 50-100 m apart) were inserted permanently 15 cm into the peat five months before the start of the flux monitoring.
Peat oxidative decomposition collected continuously in between October 2016 to May 2021 at 30 minutes frequency with four replicates.
For peat physical and chemical properties of the surface layer (0 - 50 cm), four plots in each of the Acacia plantation and degraded site, and three plots in the intact site within the eddy covariance flux footprint (200 - 1,000 m distance from each tower location). Peat samples for bulk density, pH and ash content were collected in September 2017, February 2019 and September 2019 in the intact site, in June 2017, January 2018, October 2018, and February 2019 in the degraded site and June 2017, February 2018, October 2018, February 2019 and October 2019 in the Acacia plantation. Samples for soil carbon, nitrogen, nitrate, ammonium content were collected in August 2020 and October 2021 for all sites.

Data exclusions

Following standard eddy covariance quality control criteria, we applied quality controls to remove low-quality eddy covariance measurements, as is done for eddy covariance measurements (details are in the manuscript). We removed oxidative peat decomposition measurements with negative values, fluxes with coefficients of regression of < 0.9, or values that were extreme outliers (≥ 99th percentile).

Reproducibility

Not applicable due to the nature of the research

Randomization

Sampling plots and collars for soil N2O fluxes monitoring and plots for peat soil sampling were located randomly around each eddy covariance tower within flux footprint i.e. 200 - 1000 m radius from eddy covariance tower.

Blinding

Not applicable due to the nature of the research

Did the study involve field work? ☒ Yes ☐ No

Field work, collection and transport

Field conditions

This study was conducted in the Kampar Peninsula (Sumatra, Indonesia), an ombrogenous tropical peatland of around 700,000 ha that largely formed within the past 5,100 years. The base of the peatland is grey marine clays over which peat varies from approximately 3 m deep near the river boundaries, to over 11 m in the center of the approximately 60-km-wide and > 100-km-long peat dome, with an average depth of 8 m. The peninsula experiences a humid tropical climate with the average monthly air temperature ranging from 26 to 29 °C. The variability in rainfall is influenced by monsoonal processes combined with El Niño-Southern Oscillation (ENSO) and Indian Ocean Dipole (IOD). In general, the El Niño and positive IOD occur sequentially, with the positive IOD peaking a few months after the El Niño, exerting a strong combined effect on regional rainfall patterns. The average annual rainfall for the past eight years (2014-2021, with El Niño in 2015, La Niña in 2017 and a major positive IOD combined with an El Niño event in 2019) is 1,772 ± 201 mm. Rainfall varies seasonally with two annual peaks, in November-December and another in March-April.

Location

Acacia plantation. Latitude : 0° 30′ 57.221″ N; Longitude : 102° 2′ 11.090″E; vegetation-canopy height : 17 ± 6 m; peat depth : 7 ± 0.8 m; groundwater level : -0.65 ± 0.17 m.
Degraded peat swamp forest. Latitude : 0° 41′ 58.169″ N; Longitude : 102° 47′ 35.898″ E; vegetation-canopy height: 19 ± 6 m; peat depth : 8.4 ± 1.0 m; groundwater level : -0.69 ± 0.18 m.
Intact peat swamp forest. Latitude : 0° 23′ 42.735″ N; Longitude: 102° 45′ 52.382″E; vegetation-canopy height : 32 ± 6 m; peat depth : 9 ± 1.0 m; groundwater level : -0.24 ± 0.22 m.

Access & import/export

No sample import/export efforts have been made in this study. The establishment and operation of the eddy covariance towers and associated data collection were funded and approved by Asia Pacific Resources International Ltd (APRIL) and Riau Ecosystem Restoration (RER). Acacia plantation site is managed by APRIL, the degraded site was an unmanaged area which located in the Acacia plantation boundary, whereas the intact site is located in the RER conservation area.

Disturbance

No disturbance has been caused due to this study.

Reporting for specific materials, systems and methods

We require information from authors about some types of materials, experimental systems and methods used in many studies. Here, indicate whether each material, system or method listed is relevant to your study. If you are not sure if a list item applies to your research, read the appropriate section before selecting a response.

Materials & experimental systems

n/a	Involved in the study
☒	☐ Antibodies
☒	☐ Eukaryotic cell lines
☒	☐ Palaeontology and archaeology
☒	☐ Animals and other organisms
☒	☐ Clinical data
☒	☐ Dual use research of concern

Methods

n/a	Involved in the study
☒	☐ ChIP-seq
☒	☐ Flow cytometry
☒	☐ MRI-based neuroimaging
