# Supplementary material for: Net greenhouse gas balance of fibre wood plantation on peat in Indonesia
Source: Nature. 2023 Apr 5;616(7958):740–6. doi: 10.1038/s41586-023-05860-9 (PMC10132972; doi:10.1038/s41586-023-05860-9)
Supplement: Supplementary file 3 — Peer Review File [file 41586_2023_5860_MOESM3_ESM.pdf]

Peer Review File

Manuscript Title: Net greenhouse gas balance of fiber wood plantation on peat in Indonesia

Reviewer Comments & Author Rebuttals

Reviewer Reports on the Initial Version:

Referees' comments:

Referee #1 (Remarks to the Author):

This study reports on a wide range of biogeochemical measurements made from an Acacia plantation on peat soils and compares the results with previously published data from degraded peatland soils, and intact peatland. There is a wealth of data reported for each site, including eddy covariance data for CO2 and CH4, a preliminary water balance, and peatland soil characteristics as well as harvest information for the plantation site. These data are extremely valuable. That said, I wondered about the ability to generalize from these results. There is only one replicate of each site, and it appears that the sites may actually lack independence as the degraded site is really an edge location. This may also be true of the intact site; it was unclear from the text. It is also unclear if it is appropriate to assume that the three sites all had the same starting condition and are thus linearly related as the text implies. A broader survey of site characteristics would help confirm if this was the case.

I thought it was interesting that the mineral N concentrations, especially nitrate, were so high. This suggests that the sites are well aerated. How frequently and at what depths were these measurements made? I did not see details of the soil biogeochemistry measurements. This is also true for soil C.

N2O was measured within 2 km of the flux towers, which seems to be very far away from the rest of the measurements. Were the measurements always in the primary footprint of the towers? How frequently were the N2O measurements made? The statement is made that ~1000 measurements were made, but now how they were distributed across treatments or time.

The paper is generally well written although it would benefit from a tighter organization and focus on the primary results.

Referee #2 (Remarks to the Author):

Deshmukh et al. present an interesting and unique multiyear dataset of ecosystem-scale greenhouse gas (GHG) fluxes from an Acacia crassicarpa plantation, degraded forest and intact forest within the same peat landscape to assess the impacts of land-cover change on GHG fluxes in Sumatra, Indonesia. The measurements represent the first full plantation cycle GHG budget on peatlands worldwide. The primary findings are that the GHG emissions from the Acacia plantation over a full

plantation cycle were two times higher than those from the intact forest, but less than the current IPCC Tier 1 emissions factor. Given the challenges and cost of conducting these types of measurements (including N2O fluxes, fluvial carbon export, and biomass carbon loss), this is truly a unique and very rich dataset, particularly in an underrepresented region of the world. The findings of this work are not only applicable to scientists, but also have strong policy implications, and are of relevance to better predicting future climate change.

While the dataset is unique and the findings are of interest to a broad audience, I would encourage the authors to more clearly describe (and consider the assumptions) how uncertainty was calculated. Furthermore, I have found a potential error in the total GHG balance calculations. I would also use RF/ANN gap-filling for methane fluxes. These major comments, along with some more minor comments, are outlined below. However, once these issues are addressed, I believe this manuscript shows strong potential for publication in Nature.

Major comments:

1) Uncertainty estimates:

In the paper you describe gap-filling and random uncertainty, and uncertainty due to the friction velocity threshold. You also note that total uncertainty in fluxes was calculated using the law of propagation of errors. Do the total errors represent gap-filling, random, and ustar uncertainty? Or only random and ustar uncertainty (e.g., https://github.com/bgctw/REddyProc/blob/master/vignettes/aggUncertainty.md)?
Can you elaborate more on total uncertainty? Furthermore, in Extended Data Table 3, you note that annual estimates are presented with the standard deviation from different gap-filling approaches. Do you mean the spread of the 20 modelled ANN/RF procedures? Or the spread between the ANN/RF/MDS gap-filling values? It doesn't seem like you include random and ustar uncertainty there? It would be helpful to better described how uncertainties in annual sums were calculated.

Furthermore, you note that random uncertainty is estimated using the method of Finklestein and Sims. Why not use the random uncertainty from the MDS standard deviation as described here: https://github.com/bgctw/REddyProc/blob/master/vignettes/aggUncertainty.md? As noted by El madany et al. 2018, "the MDS uncertainties include information on spatial variability (given changes in the footprints between half hours), on temporal variability (given the +/−7 days sampling window around an observation), and to a certain degree also variability in meteorological conditions (which should be relatively small given the narrow bin widths)" while the Finklestein and Sims method "is only based on half hourly turbulent time series (e.g. vertical wind speed and CO2) and their auto- and cross-correlation, hence containing no, or only very limited information about spatial, temporal, or meteorological variability." Since you already have the MDS estimate, I would suggest using that as it is more comprehensive.

Related to uncertainty estimates, how did you conduct the ustar filtering. On Line 735 you note "After a set of quality controls69-71,…" does this include ustar filtering? How was ustar filtering done? Also, for the ustar uncertainty, why not run the full ustar distribution for u* uncertainty (e.g.,

https://github.com/bgctw/REddyProc/blob/master/vignettes/aggUncertainty.md?)

2) CH_4 flux gap-filling: Why weren't ANN & RF applied to CH_4 flux as well? Recent papers (e.g., Kim et al. [Gap-filling approaches for eddy covariance methane fluxes: A comparison of three machine learning algorithms and a traditional method with principal component analysis] and Irvin et al. [Gap-filling eddy covariance methane fluxes: Comparison of machine learning model predictions and uncertainties at FLUXNET-CH4 wetlands]) advocate for using RF over MDS.

3) Total GHG budgets (e.g., Figure 2 and extended data table 4): Going back to Neubauer and Megonigal, 2015, they engineered the SGWP metric to account for C seq and not C uptake (it's not EC-centric in this way). So instead of a balance between net CO_2 uptake and CH_4 emitted, it needs to be a balance between C sequestered and CH_4 emitted. So for example, for your sites, based on the excerpt from the paper below, you would need to remove the C-CH_4 component lost from the net NEE uptake and remove fluvial C export and Biomass C loss (all in units of C). This doesn't change the numbers drastically, but I think it would be important to do if using the SGWP approach. Attached I provide an example calculation for the Intact peatland (hopefully I didn't make any mistakes – but regardless, I would make sure to double check your numbers and consider the points from Neubauer and Megonigal, 2015 below).

Neubauer and Megonigal, 2015 quote: "Any reader who wishes to apply these SGWP and SGCP values to determine whether a site is a source or sink of greenhouse gases should be aware that these ratios are based on rates of ecosystem CO_2 sequestration, not net rates of CO_2 uptake. This decision was based on practicality, as our impression is that more ecosystem scientists measure C sequestration (for example, by quantifying wood accumulation and/or using 137Cs and 210Pb radiodating techniques in accreting soils) than develop robust annual estimates of net ecosystem CO_2 exchange (for example, by using flux chambers or eddy covariance methods)."

Minor points:

Abstract
1. In the abstract, I would suggest including a first sentence highlighting the importance of tropical peatlands in the global C cycle.
2. In the abstract, in the last sentence, can you elaborate on who/what is impacted by your findings (eg., implications for the climate system? implications for C credits? Implications for national GHG inventories?)

3. Line 72 – can you provide a range of the uncertainty in the text for readers who are less familiar with those estimates?

4. Line 142: I would suggest 'The substantial net CO_2 emissions' (rather than The substantial NEE-CO_2 during…)

5. Line 154: Usually NEP is defined as -NEE. I know you clarify that you use the same sign convention for NEE and NEP, but that might cause some confusion to readers from different fields.

6. Lines 185-186: I would suggest 'net CO_2 emissions' or 'net CO_2 uptake' rather than just 'CO_2 emissions' or 'CO_2 uptake'. And the same elsewhere in the manuscript.

7. Lines 760 – You note "Therefore, we used the GWL as an environmental factor for the look-up table to derive the daytime Reco data using the MDS gap-filling algorithm". Did you include any other variables such as soil temperature or air temperature?

8. For Extended data table 3 (and others) - For 'measurement period', please specify month/year (as to not confuse it with month/day).

Attachment:

	**Net GHG budget from the paper**		**Net GHG budget as proposed by Neubauer and Megonigal, 2015**		
NEE	15.3	tCO2 h-1 yr-1	C sequestration (NEE - CH4-C - Fluvial C)	13.97	tCO2-e h-1 yr-1
FCH4	3.43	tCO2-e h-1 yr-1	FCH4	3.43	tCO2-e h-1 yr-1
Fluvial C	1.1	tCO2-e h-1 yr-1	Fluvial C	1.1	tCO2-e h-1 yr-1
soil N2O	0.08	tCO2-e h-1 yr-1	soil N2O	0.081	tCO2-e h-1 yr-1
**Total**	19.91	tCO2-e h-1 yr-1	**Total**	18.59	tCO2-e h-1 yr-1

Referee #3 (Remarks to the Author):

General
This extensive and timely work using Eddy Covariance (EC) method is very much welcome by the scientific community. The authors described the long-term experiments involving natural topical peat swamp forest (PSF), degraded/logged-over forests, and industrial plantation established from PSF conversion. The net GHG exchange results provide useful insights and new understanding of a complex process in tropical environment.
However, I have a few concerns that need clarity/ explanation around the flux change approaches in comparing with the IPCC guidelines. Firstly, being the most comprehensive account of the process, it is utmost logical to recognize numerous ground-based/static chamber studies in the background, which were considered to be overestimating by the authors.

Secondly, methodological wise, while this is not the first to implement EC approach the description of the experimental setup is lacking necessary background information associated with existing natural condition, given the extent of the flat landscape etc.

Thirdly, it is mentioned throughout the text that the accounting of GHG flux in the plantation system is a full cycle, however, it is not clear which cycle it was. This is just fair as soil C-stock in the second

and following cycles would be logically much less event to compare with logged-over PSF. Otherwise the paper almost appears like defending plantation and approve that conversion is not too bad.

Specific
I found C-store is used. Probably C-input, C-losses and C-gains may be consistently hyphenated
L.49: in terms of simulated C accumulation using modified Holocene Peat Model for the tropics, you may want to consider: https://onlinelibrary.wiley.com/doi/10.1111/gcb.12672

L.79: relevant reference(s) are needed to support the statement

L.87: knowing that IPCC Guidelines are designed for GHG Inventory, I am not sure if it is appropriate to comment of its consistency here. Every new (emission factor) number may be contributed as refinement and used at project based GHG accounting. Having said that, it does not mean that the guidelines fails to proof one of its principles, consistency.

L.117-119: even so this is not the first EC method applied. It might be prudent to describe the extent and completeness of the experiments in representing the ecosystem while recognizing not being the first

L.167: please remove the word “emission”

L.168-177: to make the budget complete, this story of GWL effect on emission may be added at the Acacia plantation, in addition to NEE story.

L.178-189: again, it is obscure to jump into this claim from policy perspectives. I would suggest toning down the statement and focus on the science and keep it neutral.

L.198: spell out “LT”. Also, evapotranspiration cannot be neglected, especially is dry season. Aerodynamic process (wind removal) can be VERY significant I such a rough surface like forest stands. To make the story complete, I suggest plotting the contrasting daily ETP in wet and dry seasons. At least in the Extended information/data section.

Since ETP represent (potential) atmospheric demand, mifgt be worth plotting the actual evapotranspiration (ETA) and see what the actual effects on GWL and associated fluxes were.

L.209-216: It depends on the phase of IOD. In positive phase, IOD would enhance Southern Oscillation Index (SOI), which means worsening drought. But in negative phase SOI will be diminished or cancelled (see also comment on L.525-530 below).

L.353-356: sound cryptic. It would be appropriate to elaborate what is meant by compatible.

L.525-530: I do not think it is true that ENSO and IOD affect interannual variability of the study area. Interdecadal variability probably true but need double check with the existing literature. For interannual variability I would suggest considering monsoonal processes.

L.539 onward: it may be useful to have a background how Acacia plantation was established. I believe there are ample information on story of biodiversity losses, soil compaction, and canalization associated with such a large-scale pristine peat swamp forest conversion. Carbon and nutrient cycling would have been significantly affected in the first place.

L.543: the word ‘ensuring” sounds like a special effort has been made to select the site. In fact, it naturally exists, probably throughout the peninsula. Please be factual.

L.881: remove the first “2002”.

Author Rebuttals to Initial Comments:

Dear Reviewers,

We thank you for encouraging and positive assessments and for providing thoughtful and constructive suggestions to improve the quality of the paper. We hope that the revised manuscript has addressed all of the concerns raised satisfactorily. Our point-by-point response is shown below, with the reviewer's comments quoted first (black text), followed by our response (blue text) and blue texts in *italics* for the actual changes to the paper with the lines in brackets indicate where the corrections have been applied in the revised manuscript.

Referee #1 (Remarks to the Author):

This study reports on a wide range of biogeochemical measurements made from an Acacia plantation on peat soils and compares the results with previously published data from degraded peatland soils, and intact peatland. There is a wealth of data reported for each site, including eddy covariance data for CO2 and CH4, a preliminary water balance, and peatland soil characteristics as well as harvest information for the plantation site. These data are extremely valuable. That said, I wondered about the ability to generalize from these results. There is only one replicate of each site, and it appears that the sites may actually lack independence as the degraded site is really an edge location. This may also be true of the intact site; it was unclear from the text. It is also unclear if it is appropriate to assume that the three sites all had the same starting condition and are thus linearly related as the text implies. A broader survey of site characteristics would help confirm if this was the case.

We thank the reviewer for the positive appraisal of the research, particularly in the context of wealth of data in this study and their value to improve knowledge of the GHG balance in tropical peatland ecosystems.

We accept the point made by the reviewer about the challenges of generalizing from our sites, and have sought to acknowledge this in the manuscript (revised text below), and to place our results in the context of comparable data, to the extent that such data exist (e.g. *Fig. 3, Extended Data Fig 5*). On the other hand, as the reviewer notes, there are few if any similarly comprehensive studies of GHG fluxes using eddy covariance technique in tropical peatlands – so we believe that our study offers unique insights which (with acknowledged caveats) should advance our understanding of these vitally important ecosystems and lead to more measurements from this globally important carbon-rich ecosystem.

We have clarified these points as follows:

"Our results should not be extrapolated to other agriculture on peat in the region (e.g., sago, oil palm, rubber plantations, etc.), or to other tropical peatlands, such as those of the Amazon and Congo basins, since they have different rainfall regimes, vegetation and peat-formation histories. Nevertheless, the strong linear relationship between CO_2 flux and GWL shown in Fig. 3a does suggest that where mean annual GWL is known,

the peatland CO_2 balance can be predicted with some degree of confidence. This is in line with work[45,47] on high-latitude peatlands suggesting that GWLs are more important than local climate or other management factors." (Lines 286 - 293)

The point about edge locations is well made and we have addressed this within the site descriptions and footprint analysis for the eddy covariance data. We have clarified the edge and boundary effect points in the degraded and intact peatland in the Methods as follows:

"*The second EC tower is located on the boundary of the degraded peatland and Acacia plantation (Fig. 1). To represent only the degraded peatland, half-hourly measurements in which the prevailing wind came from the plantation site (90 to 270°) were excluded as is commonly done in EC studies[54].*" (Lines 544 - 547)

"*An integrated climatologic footprint analysis[53] indicated that approximately 80% of the fluxes were derived within 1,000 m in the upwind direction (Fig. 1), and thus originated from intact forest with neither logging nor canal-construction activity[17]. Some long-term regional effects of hydrological management of surrounding plantations cannot be ruled out, but a previous analysis suggested that the strongest effects occurred within 300 m of the plantation boundary[21], and recent multivariate analysis indicates that subsidence in the interior forest is independent of distance from plantation canals[22]. The nearest active plantation is 3.5 km from the flux tower and well outside of the flux footprint. Further, to avoid any possible boundary effect and associated bias, measurements from a wind direction between 78 and 191° were excluded in this study (Fig. 1).*" (Lines 561 - 571)

We have clarified "the same starting condition" point as follow:

"*The relatively close proximity of the Acacia plantation, intact peatland, and degraded peatland sites (Fig. 1) within the same peat landscape avoids potentially confounding variables such as differences in past natural succession[55] and peat formation[8].*" (Lines 578 - 581)

I thought it was interesting that the mineral N concentrations, especially nitrate, were so high. This suggests that the sites are well aerated. How frequently and at what depths were these measurements made? I did not see details of the soil biogeochemistry measurements. This is also true for soil C.

Yes, we agree with the referee 1. The measured groundwater levels clearly indicate that the sites were well aerated, including an intact peatland particularly in the dry seasons (*Extended Data Table 2*).

We have added text about peat sampling for physical and chemical properties in the Methods as follows:

"

For peat physical and chemical properties of the surface layer (0 - 50 cm), four plots in each of the Acacia plantation and degraded site, and three plots in the intact site were randomly selected within the EC flux footprint (200 - 1,000 m distance from each tower location, Fig. 1). At each plot, ten sub-samples within 200 m radius were composited. Peat samples for bulk density, pH and ash content were collected in September 2017, February 2019 and September 2019 in the intact site, in June 2017, January 2018, October 2018, and February 2019 in the degraded site and June 2017, February 2018, October 2018, February 2019 and October 2019 in the Acacia plantation. Samples for soil C, N, nitrate, ammonium content were collected in August 2020 and October 2021 for all sites." (Lines 651 - 660)

N2O was measured within 2 km of the flux towers, which seems to be very far away from the rest of the measurements. Were the measurements always in the primary footprint of the towers? How frequently were the N2O measurements made? The statement is made that ~1000 measurements were made, but now how they were distributed across treatments or time.

For soil N_2O emissions measurement, four plots in each of the *Acacia* plantation and degraded site, and three plots in the intact were randomly selected within the EC flux footprint (200 – 1,000 m distance from tower location, *Fig. 1*). The measurements were conducted on a bi-monthly basis. As suggested, we have added number of N_2O flux chamber measurements and their distribution in time across treatments and in *Extended Data Fig. 4*. Further, we have amended texts in Supplementary Method 2 as follows:

"*Soil N_2O fluxes were measured using manual flux chamber technique[7]. Four plots in each of the Acacia plantation and degraded site, and three plots in the intact were randomly selected within EC flux footprint (200 - 1,000 m distance from the EC tower location Fig. 1).*" (Lines 976 - 979)

"A total 308, 337 and 328 flux measurements were made between December 2019 and March 2022 for the plantation site, between July 2019 and March 2022 for the degraded peatland, and between June 2019 and February 2022 for the intact peatland on a bi-monthly basis, covering the dry and wet seasons to represent the variation in the GWL and soil temperature." (Lines 996 - 1001)

The paper is generally well written although it would benefit from a tighter organization and focus on the primary results.

Thank you for the positive assessment. We have gone through the revised manuscript and tried to tighten it up where possible, in line with the Editorial guidelines.

Referee #2 (Remarks to the Author):

Deshmukh et al. present an interesting and unique multiyear dataset of ecosystem-scale greenhouse gas (GHG) fluxes from an Acacia crassicarpa plantation, degraded forest and intact forest within the same peat landscape to assess the impacts of land-

cover change on GHG fluxes in Sumatra, Indonesia. The measurements represent the first full plantation cycle GHG budget on peatlands worldwide. The primary findings are that the GHG emissions from the Acacia plantation over a full plantation cycle were two times higher than those from the intact forest, but less than the current IPCC Tier 1 emissions factor. Given the challenges and cost of conducting these types of measurements (including N2O fluxes, fluvial carbon export, and biomass carbon loss), this is truly a unique and very rich dataset, particularly in an underrepresented region of the world. The findings of this work are not only applicable to scientists, but also have strong policy implications, and are of relevance to better predicting future climate change.

Thank you for the positive assessment.

While the dataset is unique and the findings are of interest to a broad audience, I would encourage the authors to more clearly describe (and consider the assumptions) how uncertainty was calculated. Furthermore, I have found a potential error in the total GHG balance calculations. I would also use RF/ANN gap-filling for methane fluxes. These major comments, along with some more minor comments, are outlined below. However, once these issues are addressed, I believe this manuscript shows strong potential for publication in Nature.

Thanks for assessing the manuscript positively and providing inputs for further improvement. We have clarified the calculations of uncertainties and total GHG balance and applied Random Forest and ANN gap-filling methods for net ecosystem CH_4 exchanges, as discussed further below.

Major comments:

1) Uncertainty estimates:

In the paper you describe gap-filling and random uncertainty, and uncertainty due to the friction velocity threshold. You also note that total uncertainty in fluxes was calculated using the law of propagation of errors. Do the total errors represent gap-filling, random, and ustar uncertainty? Or only random and ustar uncertainty (e.g., https://github.com/bgctw/REddyProc/blob/master/vignettes/aggUncertainty.md)? Can you elaborate more on total uncertainty? Furthermore, in Extended Data Table 3, you note that annual estimates are presented with the standard deviation from different gap-filling approaches. Do you mean the spread of the 20 modelled ANN/RF procedures? Or the spread between the ANN/RF/MDS gap-filling values? It doesn't seem like you include random and ustar uncertainty there? It would be helpful to better described how uncertainties in annual sums were calculated.

Furthermore, you note that random uncertainty is estimated using the method of Finklestein and Sims. Why not use the random uncertainty from the MDS standard deviation as described here:
https://github.com/bgctw/REddyProc/blob/master/vignettes/aggUncertainty.md? As

noted by El madany et al. 2018, “the MDS uncertainties include information on spatial variability (given changes in the footprints between half hours), on temporal variability (given the +/−7 days sampling window around an observation), and to a certain degree also variability in meteorological conditions (which should be relatively small given the narrow bin widths)” while the Finklestein and Sims method “is only based on half hourly turbulent time series (e.g. vertical wind speed and CO2) and their auto- and cross-correlation, hence containing no, or only very limited information about spatial, temporal, or meteorological variability.” Since you already have the MDS estimate, I would suggest using that as it is more comprehensive.

Related to uncertainty estimates, how did you conduct the ustar filtering. On Line 735 you note “After a set of quality controls69-71,…” does this include ustar filtering? How was ustar filtering done? Also, for the ustar uncertainty, why not run the full ustar distribution for u* uncertainty (e.g., https://github.com/bgctw/REddyProc/blob/master/vignettes/aggUncertainty.md?)

We apologize for the misunderstanding. We did indeed calculate total uncertainty from random error in measurement, friction velocity quality control criteria, and gap-filling approach in line with the uncertainty approach outlined in Pastorello *et al*. (2020) used in the global Fluxnet2015 dataset and implemented in the OneFlux software package. We also applied u* threshold filtering as a quality control criterion following Wutzler *et al*. (2018) as recommended by the reviewer. We have revised the text in Methods for clarity as follows:

“*The flux random uncertainty was calculated following ref*[80]*. The standard deviation of three different flux values derived from friction velocity thresholds of 5th, 50th and 95th percentiles were applied as an uncertainty due to the friction velocity threshold using the REddyProc package*[77]*. The gap-filling flux uncertainty was calculated from the standard deviation of the MDS procedure*[77]*. Averages of the 20 ANN and RF modelled values were used to fill gaps and the standard deviation was used to quantify the uncertainty due to gap-filling. The total uncertainty in EC measurements of CO_2, CH_4 and evapotranspiration included gap-filling, random and friction velocity uncertainty*[81]*. Annual estimate of CO_2, CH_4, evapotranspiration and GHG balance includes total uncertainty calculated using the law propagation of errors.*” (Lines 737 - 746)

2) CH4 flux gap-filling: Why weren’t ANN & RF applied to CH4 flux as well? Recent papers (e.g., Kim et al. [Gap-filling approaches for eddy covariance methane fluxes: A comparison of three machine learning algorithms and a traditional method with principal component analysis] and Irvin et al. [Gap-filling eddy covariance methane fluxes: Comparison of machine learning model predictions and uncertainties at FLUXNET-CH4 wetlands]) advocate for using RF over MDS.

Based on the reviewer’s comments, we undertook Random Forest and ANN gap-filling methods for the net ecosystem CH_4 exchanges. We are grateful to the reviewer for this suggestion, which is now fully incorporated in the analysis, and has strengthened our findings.

We have added the text as follows:

"*We gap-filled both low-quality and missing data, as is commonly done in EC studies*[16,42,56,71-75]*. Following re*[16]*, we applied three gap-filling approaches for CO_2: (1) marginal distribution sampling (MDS)*[56,76]*, (2) artificial neural network (ANN)*[73] *and (3) random forest (RF)*[74] *separately for the daytime (06:00 - 18:00 local time) and the nighttime (18:00 - 06:00 local time) data.*" (Lines 707 - 711)

"We applied the above gap-filling approaches for evapotranspiration and CH_4 as well. For evapotranspiration, we applied net radiation instead of PPFD during the daytime, whereas net radiation and the VPD were excluded during the nighttime. After gap-filling, we corrected the daily evapotranspiration for the energy imbalance using net radiation, sensible heat and latent heat as described in ref[79]*."* (Lines 727 - 731)

"For CH_4, we used GWL, VPD, air temperature, friction velocity, latent heat flux, sensible heat flux, atmospheric pressure and global radiation in the daytime and latent heat flux and global radiation were excluded in the nighttime for ANN and RF. For MDS, we used latent heat flux, GWL and air temperature during the daytime and GWL, air temperature and soil temperature during the nighttime." (Lines 732 -736)

3) Total GHG budgets (e.g., Figure 2 and extended data table 4): Going back to Neubauer and Megonigal, 2015, they engineered the SGWP metric to account for C seq and not C uptake (it's not EC-centric in this way). So instead of a balance between net CO2 uptake and CH4 emitted, it needs to be a balance between C sequestered and CH4 emitted. So for example, for your sites, based on the excerpt from the paper below, you would need to remove the C-CH4 component lost from the net NEE uptake and remove fluvial C export and Biomass C loss (all in units of C). This doesn't change the numbers drastically, but I think it would be important to do if using the SGWP approach. Attached I provide an example calculation for the Intact peatland (hopefully I didn't make any mistakes – but regardless, I would make sure to double check your numbers and consider the points from Neubauer and Megonigal, 2015 below).

Neubauer and Megonigal, 2015 quote: "Any reader who wishes to apply these SGWP and SGCP values to determine whether a site is a source or sink of greenhouse gases should be aware that these ratios are based on rates of ecosystem CO2 sequestration, not net rates of CO2 uptake. This decision was based on practicality, as our impression is that more ecosystem scientists measure C sequestration (for example, by quantifying wood accumulation and/or using 137Cs and 210Pb radiodating techniques in accreting soils) than develop robust annual estimates of net ecosystem CO2 exchange (for example, by using flux chambers or eddy covariance methods)."

Thank you for the detailed review of the SGWP calculation. In our study, the studied ecosystems were sources of CO_2, CH_4 and N_2O. CO_2 emissions from our studied ecosystems have a warming effect on the atmosphere during the measurement period and thus all SGWP values are additive following the outline here. We believe that there

no further change is required in our total GHG budgets, but if we have misunderstood the reviewers' point here we would be happy to revisit this.

Minor points:

Abstract

1. In the abstract, I would suggest including a first sentence highlighting the importance of tropical peatlands in the global C cycle.

The following sentence has been added:

"*Tropical peatlands cycle and store globally significant amounts of carbon in their soil and biomass.*" (Lines 27 - 28)

2. In the abstract, in the last sentence, can you elaborate on who/what is impacted by your findings (eg., implications for the climate system? implications for C credits? Implications for national GHG inventories?)

We have added the text on implications of our findings as follows:

"*Our results should help to reduce the uncertainty in the estimation of GHG emissions from globally important ecosystems, provide estimate of the impact of land-use change on tropical peat, and develop science-based peatland management practices as nature-based climate solutions that help to minimize GHG emissions.*" (Lines 41 - 44)

3. Line 72 – can you provide a range of the uncertainty in the text for readers who are less familiar with those estimates?

We have added a range in the text as follows:

"*Existing estimates of GHG emissions from tropical peatlands continue to be debated[23,27] with large observed variability (0.04-2.79 $GtCO_2$-eq yr^{-1})[28] and resulting uncertainty[29].*" (Lines 68 - 69)

4. Line 142: I would suggest 'The substantial net CO2 emissions' (rather than The substantial NEE-CO2 during…)

We have revised the text as follows:

"*The substantial **net** CO_2 emissions during the early stage of the plantation …*" (Lines 97 - 98)

5. Line 154: Usually NEP is defined as -NEE. I know you clarify that you use the same

sign convention for NEE and NEP, but that might cause some confusion to readers from different fields.

We do not use NEP term anymore in the revised manuscript to avoid possible confusion to readers from different fields.

6. Lines 185-186: I would suggest 'net CO2 emissions' or 'net CO2 uptake' rather than just 'CO2 emissions' or 'CO2 uptake'. And the same elsewhere in the manuscript.

We have revised the text as follows:

"*Net ecosystem exchanges of CO_2 (NEE-CO_2; net gaseous CO_2 exchange between ecosystem and atmosphere) varied with plantation age; it was highest (48.4 ± 4.7 tCO_2 ha^{-1} yr^{-1}) in the first year after planting, lowest (-8.8 ± 4.5 tCO_2 ha^{-1} yr^{-1}) in the third year with highest tree growth, and then rose again to 11.7 ± 6.0 tCO_2 ha^{-1} yr^{-1} before harvesting (Extended Data Fig. 1 and Extended Data Table 3) (where positive NEE-CO_2 values indicate net CO_2 emissions and negative values indicate net CO_2 uptake)." (Lines 91 - 97)*

"*The substantial **net** CO_2 emissions during the early stage of the plantation….*" (Lines 97 - 98)

"*The results indicate that large **net** CO_2 emissions during dry seasons were not entirely balanced by relatively small **net** CO_2 uptake during the wet seasons..*." (Lines 140 - 141)

7. Lines 760 – You note "Therefore, we used the GWL as an environmental factor for the look-up table to derive the daytime Reco data using the MDS gap-filling algorithm". Did you include any other variables such as soil temperature or air temperature?

Yes, we used groundwater level, air temperature and soil temperature for the look-up table to derive the daytime R_{eco} data using the MDS gap-filling algorithm.

We have clarified this point in Methods as follows:

"*Therefore, we used the GWL, air temperature, and soil temperature as environmental factors for the look-up table to derive the nighttime CO_2 exchanges using the MDS gap-filling algorithm*." (Lines 718 - 721)

8. For Extended data table 3 (and others) - For 'measurement period', please specify month/year (as to not confuse it with month/day).

We agree - we have amended texts with all four digits of the calendar year in *Extended Data Table 2, 3* and *5* to avoid any possible confusion. e.g. "*October 2016 – September 2017*"

Referee #3 (Remarks to the Author):

General

This extensive and timely work using Eddy Covariance (EC) method is very much welcome by the scientific community. The authors described the long-term experiments involving natural topical peat swamp forest (PSF), degraded/logged-over forests, and industrial plantation established from PSF conversion. The net GHG exchange results provide useful insights and new understanding of a complex process in tropical environment.

Thank you for the positive assessment.

However, I have a few concerns that need clarity/ explanation around the flux change approaches in comparing with the IPCC guidelines. Firstly, being the most comprehensive account of the process, it is utmost logical to recognize numerous ground-based/static chamber studies in the background, which were considered to be overestimating by the authors.

As the reviewer notes, a substantial number of chamber- and subsidence-based studies that have tried to estimate the carbon and GHG balance of tropical peatlands, we refer to these studies in the context of IPCC Tier 1 emission factors in our introduction, and subsequently compare our results with those obtained using similar (EC) and different (chamber, subsidence) methods in *Fig. 3* and associated discussion. Based on this comment we have added some additional text here noting the discrepancy between CO_2 fluxes obtained by the different methods – we prefer not to enter into a detailed discussion of the pros and cons of each approach, or to suggest that other methods are intrinsically flawed, but we have noted that the differences we observed here are not unique to tropical peatlands, but does exist for the northern peatlands also as at the following figure.

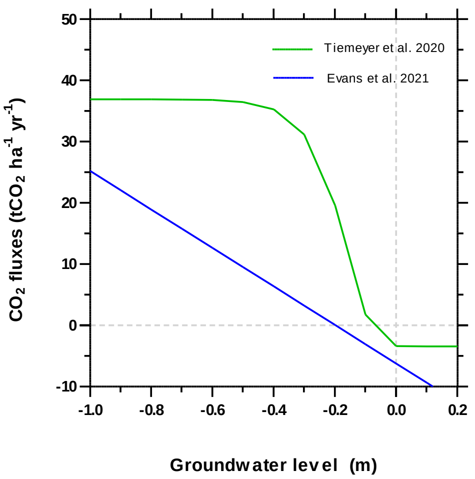

We have added the text as follows:

"*At present, the Intergovernmental Panel on Climate Change (IPCC) Tier 1 CO_2 emission factor (EF)*[23] *for short-rotation tree plantations on tropical peat is entirely based on the use of short-term measurements from the 3 - 8 years after drainage using subsidence*[18] *and soil chamber*[19] *techniques. In addition, tropical peatlands emit methane (CH_4)*[24,25] *and nitrous oxide (N_2O)*[16,26]*, potent greenhouse gases (GHGs), yet assessments of the contributions made by these gases to the full peatland GHG balance are scarce*[23]*.*" (Lines 61 - 67)

"As is evident from Fig. 3a, net CO_2 fluxes and their relationships with GWL derived from EC studies are substantially lower than those obtained from chamber and subsidence studies in the same ecosystems. While the number of published EC studies from tropical peatlands remains insufficient to establish whether these differences are systematic, a similar offset is evident in CO_2 flux versus GWL relationships derived from EC data[45] *and chamber data*[46] *for high-latitude peatlands."* (Lines 275 - 281)

Secondly, methodological wise, while this is not the first to implement EC approach the description of the experimental setup is lacking necessary background information associated with existing natural condition, given the extent of the flat landscape etc.

Based on this comment and previous comments from Referee 1, we have revised the text for clarity in the Methods section. Specifically, we have added the additional information related to frequency of measurement and the flux footprint area to describe the extent and completeness of the experiments in representing the ecosystem. We have addressed this within the site descriptions and footprint analysis for the eddy covariance data – see response for Referee 1

"*Eddy covariance provides half-hourly measurements of turbulent exchanges between an entire ecosystem and the atmosphere above the vegetation canopy*[54]*. Hence, eddy covariance measurements incorporate all existing sources and uptakes that can vary significantly within an ecosystem in both space and time arising from variation in environmental conditions. Given the flat terrain (slope $< 0.05\%$), using the measured vegetation-canopy height and wind speed, the estimated 80% EC flux footprints represent an area of interest of around 1,000 m radius (Fig. 1)."* (Lines 572 - 578)

Thirdly, it is mentioned throughout the text that the accounting of GHG flux in the plantation system is a full cycle, however, it is not clear which cycle it was. This is just fair as soil C-stock in the second and following cycles would be logically much less event to compare with logged-over PSF. Otherwise the paper almost appears like defending plantation and approve that conversion is not too bad.

This is a good point and we have included in our revision. We have added more information on the establishment of the plantation (see response to comment on Line 539 below), as well as noting that our measurements cover the 4th rotation of the

plantation during 17-22 years after drainage in both the methods and at the start of the results.

"*Acacia plantation: Over a 4.7-year period encompassing one full Acacia plantation rotation (the fourth rotation during 17-22 years after drainage)*…" (Lines 88 - 89).

"*When measurements began in October 2016, the trees were already at the end of the third plantation rotation. All plantation compartments within 2-km radius around the EC tower were harvested between October 2016 and April 2017. Tree height at harvest was in the range from 19 to 24 m, determined from a vegetation survey in permanent sampling plots (20 m × 125 m) around the tower. Replanting for the fourth plantation rotation took place within two weeks after harvesting each individual compartment at a density of 1,667 trees per hectare (3 m × 2 m spacing). Five grams of chelated micronutrients per tree were applied around the seedlings during planting. All compartments within a 2-km radius of the EC tower were harvested between June and August 2021, when the average plantation age was 4.7-years, and replanting for the fifth plantation rotation took place within two weeks after harvesting."* (Lines 519 - 530)

Specific
I found C-store is used. Probably C-input, C-losses and C-gains may be consistently hyphenated

Thanks and agreed. We have replaced the term "C export" with "C-export", "C input" with "C-input", "C loss" with "C-loss", "C balance" with "C-balance", and "C content" with "C-content" as suggested.

*"A fine balance between hydrology, ecology and landscape morphology has resulted in this long-term **C-store**[6-8]. Climate and other environmental changes are, however, affecting this **C-store** as a result of warming, drying conditions and change in disturbance rates[9-16]. Particularly, decreased rainfall, increased seasonality and frequent days without rainfall are resulting in groundwater level (GWL) drawdowns[7,12,13], which cause **C-loss**[14-16]."* (Lines 47 - 52)

*"Thus, a better quantitative and process-based knowledge of how the tropical peat **C-store** responds…"* (Lines 76 - 77)

*"…including biomass **C-loss** due to plantation establishment, **C-export** in harvested wood and fluvial **C-exports**."* (Lines 82 - 83)

*"After canopy closure, the emissions from oxidative peat decomposition (Extended Data Table 3) were largely outbalanced by high rates of photosynthesis and **C-fixation** (Extended Data Fig. 1)…"* (Lines 101 - 103)

*"The **C-export** in harvested wood was 26.3 tC ha^{-1}, corresponding to 96.5 tCO_2 ha^{-1} (20.5 tCO_2 ha^{-1} yr^{-1} when annualized over the plantation rotation; Fig. 2 and Extended Data Table 4). Thus, the sum of NEE-CO_2 and **C-export** in harvested wood indicates*

that the Acacia plantation functioned as a CO_2 source of 30.0 ± 4.5 tCO_2 ha^{-1} yr^{-1} over the plantation rotation (Fig. 2 and Extended Data Table 4).” (Lines 106 - 109)

*“…a hiatus in peat genesis[33], or even **C-loss**[14-16] have been reported in response …”* (Line 146)

*“…**C-loss** and subsidence during the study period were primarily caused by droughts driven by regional climate extremes[22]. Our results suggest that even low-level or indirect human disturbance via climate change can lead to **C-loss**…”* (Lines 177 - 180)

*“**Other GHG fluxes and C-loss**”* (Line 181)

*“A previous study[36] within the same landscape reported fluvial **C-export** of 0.3 ± 0.1 and 0.5 ± 0.1 tC ha^{-1} yr^{-1} respectively in the intact and degraded peatland. Due to lack of fluvial **C-export** measurements for the Acacia plantation, we used a value of 0.4 ± 0.1 tC ha^{-1} yr^{-1} from a managed oil palm plantation in Southeast Asia[37]. Notably, fluvial **C-exports** are fairly small compared to direct CO_2 emissions. We conservatively assume that all fluvial **C-export** is ultimately emitted as CO_2 (ref[38]). The increased fluvial **C-export** from the plantation and degraded forest may be attributed to enhanced mineralization with deeper GWL[37].”* (Lines 199 – 206)

*“…the measured above- and belowground biomass **C-stock** was highest…”* (Line 207)

*“…biomass **C-losses** due to land-use change from intact peatland were*…” (Line 213)

*“Biomass **C-loss** due to plantation…”* (Line 215)

*“…the average **C-balance** of high-latitude peatlands with multi-decadal…”* (Line 241)

*“…measured a **C-input** to the peat of around*…” (Line 261)

*“This **C-input**…”* (Lines 264-265)

*“…soil **C-input** through better post-harvest residue management. Further research is needed to confirm the potential scale of increase in **C-input** that could realistically be achieved.”* (Lines 268 - 270)

*“For the Acacia plantation site, time-integrated NEE-CO_2 over the plantation rotation was combined with **C-export** in the harvested wood. Total **C-export** in harvested wood and delivered to the mill from the total footprint area of 220 ha over the average plantation age of 4.7-year was calculated using a basic density of 455 kg m^{-3} and average **C-content** of 48.2%[56-59]. The exported wood is converted into pulp products and biomass fuel for bioenergy generation. We applied the conservative assumption that all C in exported wood would be returned to the atmosphere as CO_2. Intact and degraded peatlands were considered to have had no biomass **C-export** during the study period.”* (Lines 661 - 668)

"*The biomass **C-loss** due to land-use change was calculated from above- and belowground biomass **C-stock** differences between the intact peatland and the Acacia …"* (Lines 669 - 670)

*"…reported **C-balance** over a full plantation rotation, and thus they are not in steady state with regard to CO_2 uptake and emission, making the underlying peat **C-balance** uncertain. However, naturally forested peatlands and degraded peatlands were considered to have had no biomass **C-export** during the study period."* (Lines 970 - 974)

*"…and **C-export** in harvested wood to quantify a conservative estimate of **C-input** to the peat…"* (Lines 1035 - 1036)

L.49: in terms of simulated C accumulation using modified Holocene Peat Model for the tropics, you may want to consider: https://onlinelibrary.wiley.com/doi/10.1111/gcb.12672

We are grateful for the reference; We have added the reference and text as follows:

"Over the Holocene, tropical peatlands have accumulated at least 75 Gt of carbon (C) in partially decomposed debris (woods/root/litter/leaves) under waterlogged anoxic environments[1-5]." (Lines 45 - 47)

L.79: relevant reference(s) are needed to support the statement

On revision including all reviews, this statement is no more in the revised manuscript due to space limitations, in line with the Editorial guidelines.

L.87: knowing that IPCC Guidelines are designed for GHG Inventory, I am not sure if it is appropriate to comment of its consistency here. Every new (emission factor) number may be contributed as refinement and used at project based GHG accounting. Having said that, it does not mean that the guidelines fails to proof one of its principles, consistency.

This is a fair point and we have revised the statement to just note our refinement. Following the Nature Editorial guideline on the word limitation, the statement is no more in the revised manuscript.

L.117-119: even so this is not the first EC method applied. It might be prudent to describe the extent and completeness of the experiments in representing the ecosystem while recognizing not being the first

Following the Nature Editorial guideline on the word limitation, the statement is no more in the revised manuscript. However, we have provided additional information about EC method in response to the above second comment as:

“*Eddy covariance provides half-hourly measurements of turbulent exchanges between an entire ecosystem and the atmosphere above the vegetation canopy[54]. Hence, eddy covariance measurements incorporate all existing sources and removals that can vary significantly within an ecosystem in both space and time arising from variation in environmental conditions. Given the flat terrain (slope < 0.05%), using the measured vegetation-canopy height and wind speed, the estimated 80% EC flux footprints represent an area of interest of around 1,000 m radius (Fig. 1).”* (Lines 572 - 578)

L.167: please remove the word “emission”

Thanks – we have removed “emission” at the mentioned line.

L.168-177: to make the budget complete, this story of GWL effect on emission may be added at the Acacia plantation, in addition to NEE story.

We have revised as suggested and the text as follows:

“*The observed net CO_2 emissions can be attributed to peat aeration due to a consistently deep GWL, which enhances heterotrophic respiration rates, combined with a higher soil temperature (intact site = 27.5 ± 0.5 °C vs. Acacia site = 29.3 ± 1.0 °C; Extended Data Table 2) due to both canopy-cover loss and GWL drawdown, which further boosts microbial activities and heterotrophic respiration.*" (Lines 111 - 116)

L.178-189: again, it is obscure to jump into this claim from policy perspectives. I would suggest toning down the statement and focus on the science and keep it neutral.

We are unsure what the reviewer is referring to here as Line 178-189 in the original submission were “*Coarse woody debris from fallen dead trees may also have contributed to the emissions as fallen trees do not decompose instantaneously, providing a lagged but sustained contribution to CO_2 emissions.*” We believe that this sentence does not provide directly any policy aspects.

However, we are grateful to the reviewer for this suggestion and our revised manuscript focuses on the science and tries to present results as neutrally as possible from a policy perspective.

L.198: spell out “LT”. Also, evapotranspiration cannot be neglected, especially is dry season. Aerodynamic process (wind removal) can be VERY significant I such a rough surface like forest stands. To make the story complete, I suggest plotting the contrasting daily ETP in wet and dry seasons. At least in the Extended information/data section. Since ETP represent (potential) atmospheric demand, mifgt be worth plotting the actual

evapotranspiration (ETA) and see what the actual effects on GWL and associated fluxes were.

We have spelled out "LT" to "local time" throughout the manuscript.

The eddy covariance provided actual evapotranspiration estimates. Actual evapotranspiration measurements clearly show that evapotranspiration seems negligible in the night. The diurnal variation of evapotranspiration in both dry and wet season is added as *Extended Data Fig. 3* in the revised manuscript.

To clarify this we have amended the text as follows:

"*The evapotranspiration measurements clearly indicate that actual daily evapotranspiration (4.2 mm d^{-1}) exceeded daily rainfall…*" (Lines 148 - 149)

L.209-216: It depends on the phase of IOD. In positive phase, IOD would enhance Southern Oscillation Index (SOI), which means worsening drought. But in negative phase SOI will be diminished or cancelled (see also comment on L.525-530 below).

We investigated the combined impact of the ENSO and IOD as part of another paper on regional subsidence dynamics (Evans *et al*. 2022). The two phenomena are closely linked, with strong El Niño consistently followed by a positive phase of the IOD, lagged by a few months, which together drive regional drought. A pattern of positive IOD and La Niña cancelling each other out (or vice versa) does not appear to occur. We have revised the text as follows:

"In general, the El Niño[34] and positive IOD[35] occur sequentially, with the positive IOD peaking a few months after the El Niño, exerting a strong combined effect on regional rainfall patterns[22]." (Lines 494 - 496)

L.353-356: sound cryptic. It would be appropriate to elaborate what is meant by compatible.

We apologize for the misunderstanding on "compatible" term. We have amended the text as follows:

"Using our emission factors from the intact and degraded sites, the results highlight that, despite evidence that they may now be losing C, avoided emissions from conserving all remaining intact peat swamp forests in Indonesia (2.0 × 10^6 ha) under Indonesia's nationally determined contribution[49] and emissions reduction from restoring 4.2 × 10^6 ha by 2050 under Indonesia's LCCP (Low Carbon scenario Compatible with the Paris Agreement target)[50] will avoid GHG emissions of around 160 $MtCO_2$-eq yr^{-1}.." (Line 319 - 324)

L.525-530: I do not think it is true that ENSO and IOD affect interannual variability of the study area. Interdecadal variability probably true but need double check with the existing literature. For interannual variability I would suggest considering monsoonal processes.

See preceding response regarding the connection between the ENSO and IOD. Regarding the point about interannual versus interdecadal, we believe that the former applies because there can be (and have been) multiple ENSO/IOD events of varying magnitudes within a decade. Variations in these indices are indeed closely connected to monsoonal processes so we do not believe there is any difference of view with the reviewer on this point. We have revised text as follows:

"The variability in rainfall is influenced by monsoonal processes combined with El Niño-Southern Oscillation (ENSO) and IOD[51,52]. In general, the El Niño[34] and positive IOD[35] occur sequentially, with the positive IOD peaking a few months after the El Niño, exerting a strong combined effect on regional rainfall patterns[22]." (Lines 492 - 496)

L.539 onward: it may be useful to have a background how Acacia plantation was established. I believe there are ample information on story of biodiversity losses, soil compaction, and canalization associated with such a large-scale pristine peat swamp forest conversion. Carbon and nutrient cycling would have been significantly affected in the first place.

As suggested, we have provided additional information of water management system in the Study Area to provide all site characteristics including soil physical and chemical properties, which potentially influence GHG dynamics. We amended the text as follows:

"*This involved clearance of the remaining logged forest, artificial compaction occurred during mechanical land preparation, installation of regularly spaced water management and access canals, and planting of Acacia crassicarpa, which is harvested on a 4-5 year rotation.*" (Lines 511 - 514)

"*Water management zones comprise navigable canals, typically of 12 m width and 3 m depth, also used for transportation[21]. Branch canals of 5 – 8 m width run perpendicular to these canals at a spacing of 500 – 800 m to form plantation compartments, which contain 1 m deep field drains at a spacing of 75 m[21].*" (Lines 536 - 539)

L.543: the word 'ensuring" sounds like a special effort has been made to select the site. In fact, it naturally exists, probably throughout the peninsula. Please be factual.

We amended the text as follows:

"Given the flat terrain (slope < 0.05%),…" (Line 576)

L.881: remove the first "2002".

Yes thanks – the reference no longer exist at the revised manuscript

References:

Pastorello, G., et al. The FLUXNET2015 dataset and the ONEFlux processing pipeline for eddy covariance data. *Scientific Data*, **7**, 225, (2020).

Wutzler, T. et al. Basic and extensible post-processing of eddy covariance flux data with REddyProc. *Biogeosciences* **15**, 5015–5030 (2018).

Tiemeyer, B., et al. A new methodology for organic soils in national greenhouse gas inventories: Data synthesis, derivation and application, *Ecol. Indic.*, **109**, 105838 (2020).

Evans, C.D., Peacock, M., Baird, A.J. *et al.* Overriding water table control on managed peatland greenhouse gas emissions. *Nature* **593**, 548–552 (2021).

Evans, C. D. et al. Long-term trajectory and temporal dynamics of tropical peat subsidence in relation to plantation management and climate. *Geoderma* 116100 (2022).

Reviewer Reports on the First Revision:

Referees' comments:

Referee #1 (Remarks to the Author):

The authors have addressed the concerns I had with the manuscript and I think it will make a nice contribution to the literature.

Referee #2 (Remarks to the Author):

Deshmukh et al. provided clear and comprehensive replies to my previous comments, particularly related to gap-filling and uncertainty estimates.

However, I still have a very minor concern regarding the calculation for the GHG flux estimates.
For the net GHG estimates, I realize I made a slight error, although I still believe there is a minor error in the manuscript (similar to an error we made in a paper, which we also had to correct during revisions).

The main issue is related to the NEE term. As I noted, Neubauer and Megonigal engineered the SGWP to account for C sequestration (or C loss as in your case) and not NEE (net CO_2 uptake/loss). As such, eddy covariance measured NEE does not represent true C sequestration/loss (i.e., NEE doesn’t equal the net ecosystem carbon balance). To fully capture C sequestration/loss at the site, you also need to account for the C lost as CH_4 (in addition to the fluvial C export and Biomass C loss which you already account for). As such, C sequestration/loss = NEE + CH_4-C + Fluvial C + Biomass C. This will give you the net C sequestration/loss. Below I provide the example for the intact peatland (which doesn’t include Biomass C). Then the GHG budget becomes:

GHG budget = FCO_2-eq + (FCH_4 * SGWP) + (N_2O * SGWP), where FCO_2-eq represents C sequestration/loss (i.e., NEE + CH_4-C + Fluvial C, and Biomass-C where applicable). Note: see uploaded xlsx doc for proper formatting of the table.

Net GHG budget from the paper Net GHG budget as proposed by Neubauer and Megonigal, 2015
NEE 15.3 t CO_2 h-1 yr-1 C sequestration/loss (NEE + CH_4-C + Fluvial C) 16.62 tCO_2-e h-1 yr-1
FCH_4 3.6 tCO_2-e h-1 yr-1 FCH_4 3.6 tCO_2-e h-1 yr-1
Fluvial C 1.10 tCO_2-e h-1 yr-1 Fluvial C (included in C seq/loss term now)
soil N_2O 0.08 tCO_2-e h-1 yr-1 soil N_2O 0.081 tCO_2-e h-1 yr-1
Total 19.91 tCO_2-e h-1 yr-1 Total 20.30 tCO_2-e h-1 yr-1

The main difference here is that you should account for the C contained in CH_4 when calculating C sequestration/loss. Here I calculated CH_4-C as:
79.5 kgCH_4 h-1 yr-1 = 59.3 kgCH_4-C h-1 yr-1

And then converted 59.3 kgCH_4-C h-1 yr-1 to tCO_2-e h-1 yr-1 (i.e., 0.22 tCO_2-e h-1 yr-1).

As such, now you're fully accounting for C sequestration (or in your case loss), which is what should be included in the SGWP calculation. The C contained in CH4 is very minor in this case, but can sometimes be an important component of the C budget in peatlands.

Other than this, I feel that the article is suitable for publication as is.

Attachment:

	**Net GHG budget from the paper**		**Net GHG budget as proposed by Neubauer and Megonigal, 2015**		
NEE	15.3	tCO2 h-1 yr-1	C sequestration/loss (NEE + CH4-C + Fluvial C)	16.40	tCO2-e h-1 yr-1
FCH4	3.6	tCO2-e h-1 yr-1	FCH4	3.60	tCO2-e h-1 yr-1
Fluvial C	1.1	tCO2-e h-1 yr-1	Fluvial C (now in the C seq/loss term)		tCO2-e h-1 yr-1
soil N2O	0.08	tCO2-e h-1 yr-1	soil N2O	0.08	tCO2-e h-1 yr-1
**Total**	19.91	tCO2-e h-1 yr-1	**Total**	20.08	tCO2-e h-1 yr-1

Referee #3 (Remarks to the Author):

The revised version of the manuscript turns out to be satisfactory.
It demonstrates concrete and convincing results regarding net GHG emissions in peat land-use dynamics. The piece of work clearly contributes new scientific findings on those of challenging ecosystem to work and yet very important element in GHG emission reduction efforts.

The vast field data are well handled and thoroughly evaluated using statistically sounds techniques. Uncertainties are clarified. Referencing to the relevant works has been meticulously done that demonstrate the authors comprehension on the existing works.

Overall, I recommend that this piece of important work is accepted for publication.

Author Rebuttals to First Revision:

Dear Reviewers and Editor,

We thank you for considering our manuscript for publication in Nature. Based on the suggestion from Referee #2, we have revised the GHG flux estimates, following the sustained-flux global warming potential (SGWP) from Neubauer and Megonigal (2015). Our point-by-point response is shown below, with the reviewer's comments quoted first (black text), followed by our response (blue text) and blue texts in *italics* for the actual changes to the paper with the lines in brackets indicate where the corrections have been applied in the revised manuscript.

Referees' comments:

Referee #1 (Remarks to the Author):

The authors have addressed the concerns I had with the manuscript and I think it will make a nice contribution to the literature.

We thank the reviewer for their positive and encouraging assessment.

Referee #2 (Remarks to the Author):

Deshmukh et al. provided clear and comprehensive replies to my previous comments, particularly related to gap-filling and uncertainty estimates.

We thank the Reviewer for their positive comment and careful review for further improvement and consistency.

However, I still have a very minor concern regarding the calculation for the GHG flux estimates.

For the net GHG estimates, I realize I made a slight error, although I still believe there is a minor error in the manuscript (similar to an error we made in a paper, which we also had to correct during revisions).

The main issue is related to the NEE term. As I noted, Neubauer and Megonigal engineered the SGWP to account for C sequestration (or C loss as in your case) and not NEE (net CO_2 uptake/loss). As such, eddy covariance measured NEE does not represent true C sequestration/loss (i.e., NEE doesn't equal the net ecosystem carbon balance). To fully capture C sequestration/loss at the site, you also need to account for the C lost as CH_4 (in addition to the fluvial C export and Biomass C loss which you already account for). As such, C sequestration/loss = NEE + CH_4-C + Fluvial C + Biomass C. This will give you the net C sequestration/loss. Below I provide the example for the intact peatland (which doesn't include Biomass C). Then the GHG budget becomes:

GHG budget = FCO_2-eq + (FCH_4 * SGWP) + (N_2O * SGWP), where FCO_2-eq represents C sequestration/loss (i.e., NEE + CH_4-C + Fluvial C, and Biomass-C where applicable). Note: see uploaded xlsx doc for proper formatting of the table.

Net GHG budget from the paper Net GHG budget as proposed by Neubauer and Megonigal, 2015

NEE 15.3 tCO_2 ha^{-1} yr^{-1} C sequestration/loss (NEE + CH4-C + Fluvial C) 16.62 tCO_2-eq ha^{-1} yr^{-1}

FCH_4 3.6 tCO_2-eq h^{-1} yr^{-1} FCH_4 3.6 tCO_2-e h^{-1} yr^{-1}

Fluvial C 1.10 tCO_2-e h^{-1} yr^{-1} Fluvial C (included in C seq/loss term now)

soil N_2O 0.08 tCO_2-e h^{-1} yr^{-1}soil N_2O 0.081 tCO_2-e h^{-1} yr^{-1}

Total 19.91 tCO_2-e h^{-1} yr^{-1} Total 20.30 tCO_2-e h^{-1} yr^{-1}

The main difference here is that you should account for the C contained in CH4 when calculating C sequestration/loss. Here I calculated CH_4-C as:

79.5 $kgCH_4$ h-1 yr-1 = 59.3 $kgCH_4$-C h-1 yr-1

And then converted 59.3 $kgCH_4$-C h-1 yr-1 to tCO_2-e h^{-1} yr^{-1} (i.e., 0.22 tCO_2-e h^{-1} yr^{-1}).

As such, now you're fully accounting for C sequestration (or in your case loss), which is what should be included in the SGWP calculation. The C contained in CH_4 is very minor in this case, but can sometimes be an important component of the C budget in peatlands.

Thank you for the detailed assessment of the SGWP calculation, this recommendation is now fully incorporated in the GHG flux calculation in the revised manuscript. We have amended the GHG balance estimates in *Fig. 2, Extended Data Table 4* and related texts as follows:

"The greenhouse gas emissions from the Acacia plantation over a full plantation rotation (35.2 ± 4.7 tCO_2-eq ha^{-1} yr^{-1}, average ± standard deviation) were around two times higher than those from the intact forest (20.3 ± 3.7 tCO_2-eq ha^{-1} yr^{-1}), but only half of the current IPCC Tier 1 emission factor[20] for this land-use." (Lines 36 – 40)

"If we take the measured GHG balance of the intact forest site as a reference, and treat our data from the fourth Acacia rotation as representative of longer-term conditions, then the conversion of intact site to Acacia plantation results in a long-term net increase in GHG emissions of 18.1 ± 6.0 tCO_2-eq ha^{-1} yr^{-1} (Fig. 2)." (Lines 234 – 237)

"GHG emissions at the degraded site are about 16% higher than those of the plantation site, indicating that establishment of Acacia plantation on previously degraded site could apparently result in lower long-term GHG emissions of -7.5 ± 6.5 tCO_2-eq ha^{-1} yr^{-1}, in addition to avoided emissions from bioenergy production (Fig. 2)." (Lines 311 – 315)

As already noted by the referee, since CH_4 emissions are a minor component of total GHG balances in this study, therefore, there is no change in the related discussion and/or conclusions in the paper.

Referee #3 (Remarks to the Author):

The revised version of the manuscript turns out to be satisfactory.
It demonstrates concrete and convincing results regarding net GHG emissions in peat land-use dynamics. The piece of work clearly contributes new scientific findings on those of challenging ecosystem to work and yet very important element in GHG emission reduction efforts.

The vast field data are well handled and thoroughly evaluated using statistically sounds techniques. Uncertainties are clarified. Referencing to the relevant works has been meticulously done that demonstrate the authors comprehension on the existing works.

Overall, I recommend that this piece of important work is accepted for publication.

We thank the reviewer for their positive feedback that clearly captures the significance of this work.
